# Surface electromyography evaluation for decoding hand motor intent in children with congenital upper limb deficiency

**DOI:** 10.1038/s41598-024-82519-z

**Published:** 2024-12-30

**Authors:** Marcus A. Battraw, Justin Fitzgerald, Eden J. Winslow, Michelle A. James, Anita M. Bagley, Wilsaan M. Joiner, Jonathon S. Schofield

**Affiliations:** 1 Department of Mechanical and Aerospace Engineering, University of California, Davis, CA USA; 2 Department of Biomedical Engineering, University of California, Davis, CA USA; 3 Department of Neurobiology, Physiology and Behavior, University of California, Davis, CA USA; 4 Clinical and Translational Science Center, University of California Davis Health, Sacramento, CA USA; 5 Shriners Children’s – Northern California, Sacramento, CA USA; 6 Department of Orthopaedic Surgery, University of California Davis Health, Sacramento, CA USA; 7 Department of Neurology, University of California Davis Health, Sacramento, CA USA

**Keywords:** Paediatric research, Motor control

## Abstract

Children born with congenital upper limb absence exhibit consistent and distinguishable levels of biological control over their affected muscles, assessed through surface electromyography (sEMG). This represents a significant advancement in determining how these children might utilize sEMG-controlled dexterous prostheses. Despite this potential, the efficacy of employing conventional sEMG classification techniques for children born with upper limb absence is uncertain, as these techniques have been optimized for adults with acquired amputations. Tuning sEMG classification algorithms for this population is crucial for facilitating the successful translation of dexterous prostheses. To support this effort, we collected sEMG data from a cohort of N = 9 children with unilateral congenital below-elbow deficiency as they attempted 11 hand movements, including rest. Five classification algorithms were used to decode motor intent, tuned with features from the time, frequency, and time–frequency domains. We derived the congenital feature set (CFS) from the participant-specific tuned feature sets, which exhibited generalizability across our cohort. The CFS offline classification accuracy across participants was 73.8% ± 13.8% for the 11 hand movements and increased to 96.5% ± 6.6% when focusing on a reduced set of five movements. These results highlight the potential efficacy of individuals born with upper limb absence to control dexterous prostheses through sEMG interfaces.

## Introduction

Unilateral congenital below-elbow deficiency (UCBED) is the absence of an upper limb that occurs at the anatomical region between the proximal to distal segments of the forearm^1^. To those afflicted, this condition can pose significant challenges in psychosocial and physical functioning while they interact within their daily environments^2^. The naturalistic motor control of advanced dexterous prosthesis utilizing surface electromyography (sEMG, the measurement of the residual muscle’s electrical activity) has yet to be fully investigated for children with UCBED. As these children were born never having actuated a hand, and their muscles and limbs never fully developed, prosthesis control presents with a variety of considerations that are unique from adults or other children that acquired their limb absence later in life. For example, there has been very limited study on how the muscle activity of their affected limbs may manifest especially when attempting to move the missing hand for prosthetic control purposes^3^. This presents a limitation in the effective implementation of dexterous upper limb devices and drives the need to explore, adapt, and leverage current adult-based technologies to improve the motor control possibilities for prostheses offered to children with UCBED.

Previous work has shown that children with UCBED have a degree of biological control over their affected musculature i.e., when they attempted various missing hand movements there was measurable consistency and distinguishability of sEMG muscle excitation^4^. Furthermore, through the use of an emerging prosthetic control modality, namely ultrasound, it has also been shown that children with UCBED generate distinct patterns of muscle deformation that can be classified to decode motor intent^5^. Although these results are exciting, ultrasound-based control technology is not mature or commercially available and therefore lacks a translational component. Current state-of-the-art dexterous control utilizes sEMG classification algorithms to decode motor intent and therefore drive a prosthesis. However, little work has addressed how to effectively translate the currently available sEMG classification technologies to the pediatric UCBED population.

In contrast, extensive research has been focused on identifying which sEMG features and/or classifiers are most effective in decoding hand motor intent from the static contraction activity of forearm muscles^6–10^, to name a few. Additionally, studies have also investigated feature selection and window length for early transient detection of motor intent^11–13^. However, most of this work was done with adult able-bodied individuals, and thus it is often assumed that top-performing feature sets and classifier combinations will translate effectively to those with acquired amputations^14^; however, these assumptions are often not tested rigorously in affected populations. In studies that do employ cohorts of adults with acquired amputations, classification of missing limb movements from sEMG data typically ranges from 81 to 97% depending on the hand movement, feature set, classifier, etc.^15^. In a small cohort of adults with UCBED (N = 4), these values were significantly lower when simply applying a feature set developed for those with acquired limb loss (Hudgins set) with performance in the range of 52.1% ± 15.0% accuracy for 11 missing hand movements (including rest)^16^. Moreover, a small cohort of children (N = 4, less than 18 years old) and adults (N = 3) with UCBED have been studied utilizing a commercially available sEMG classification system, again developed for adults with acquired limb amputations^3^. The children achieved classification accuracies ranging from 80% ± 16.0% for 3 degrees of freedom^3^. As of our knowledge, only these two studies^3,16^ have examined individuals with UCBED, with the notable distinction that one investigated pediatric participants^3^. Importantly, neither study systematically adjusted the feature sets and classifiers to address the unique conditions that UCBED affected muscles may present.

The aim of this study was to investigate the extent that children with UCBED exhibit unique features and classification algorithms. Furthermore, if implemented, how might sEMG classification techniques enhance prediction accuracy while maintaining low training and testing times. To accomplish this, we assessed individual features and feature set performance for 31 time domain features, 9 frequency domain features, and 9 time–frequency domain features. Top-performing feature sets were evaluated over five different classification algorithms (classifiers). We then proposed a new generalized feature set for this unique population and compared this to two feature sets commonly implemented for adults with acquired amputation; the Hudgins feature set (HDS)^17,18^ and (2) a newly established efficient feature set (EFS)^6^. We hypothesized that a unique set of algorithmic parameters (i.e., features and classifiers) and attempted hand movements could be identified that would provide an effective balance between classification accuracy and computational time.

## Methods

### Participants

Nine participants with UCBED (8 male and 1 female, mean age of 14 years ± 4.4 years) completed the experimental protocol following relevant guidelines and regulations^4^. Written informed consent and assent were obtained from participants and their legal guardians. This study received approval from the Institutional Review Board at Shriners Children’s – Northern California. Participants had varying experiences of prosthesis use in addition to a wide range of affected limb lengths and circumferences, 8–18 cm and 15–23.5 cm, respectively (Table 1). All the children that participated in this study were clinically screened to ensure no other atypical development aside from UCBED.

**Table 1:** Participant demographics.

Subject ID	Age	Sex	Affected Limb	Length (cm)	Circumference (cm)	Type of Prosthesis Used
SHR-A	20	Male	Left	13	15	PA
SHR-B	8	Male	Right	14	20	PA
SHR-C	11	Male	Right	18	18	None
SHR-D	9	Male	Left	12.5	18.5	None
SHR-E	18	Male	Right	15	21	BP†
SHR-F	16	Female	Left	11.5	23.5	PA
SHR-G	19	Male	Left	13	21.5	Myo & PA
SHR-H	14	Male	Left	8	23	BP
SHR-I	12	Male	Right	10	21	BP

*PA* passive, *BP* body-powered, *Myo* myoelectric, †Activity specific device.

### Experimental protocol

This study uses data collected from the experimental protocol outlined in our previous work on understanding the affected muscle activity of children with UCBED^4^. Accordingly, participants were first introduced to the experiment which included an overview of the hand motions they would attempt, the equipment used, an explanation of sEMG, and general goals of the experiment. Then, seven wireless Trigno Mini sEMG electrodes from a Delsys Trigno EMG Research System (Delsys, USA) were adhered circumferentially^19–21^ around the participants’ affected forearm with double-sided adhesive (except for SHR-A who had only four sEMG electrodes due to limb size constraints). Given the unique anatomy of each child’s limb difference, electrode placement was guided by palpation of the ventral side of the affected forearm to identify the region presenting with the most muscle bulk. Here the first electrode was placed with the remainder placed equidistant circumferentially from this location^19^.

**Fig. 1:**
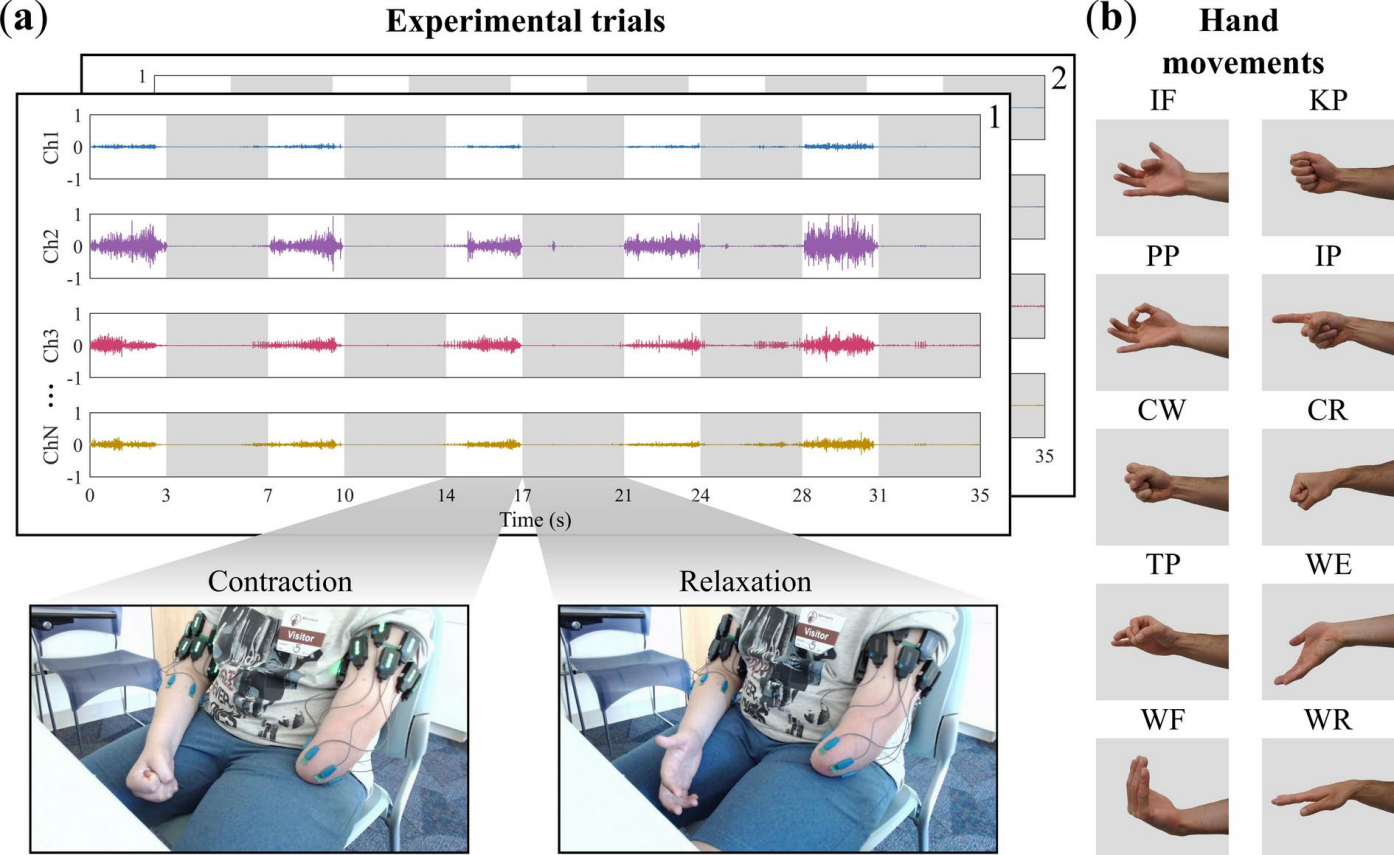
Experimental protocol. (**a**) Depicts a participant during a cylindrical wrap (CW) contraction and relaxation phase of the experiment along with the sEMG data across channels. (**b**) Displays the 10 different hand motions participants were instructed to attempt. The figure was adapted from^4^.

Participants were situated in a chair with their affected and unaffected limbs in a comfortable position by their side. Afterward, they were instructed to perform a sequence of 10 repetitions for the same missing hand movement, divided into two groups of five. Participants performed simultaneous movements with both their affected and unaffected limbs to support the mental visualization and execution of missing hand movements. The order of hand movements was randomized to reduce any potential biases. A metronome was used to ensure consistency in the repetition of movements by indicating when to execute each specified movement. Each movement was held for 3 s followed by a 4-s relax phase. A depiction of the sEMG data for the first five repetitions and a participant attempting the motion is shown in Fig. 1a. The hand movements performed represented those most commonly used during activities of daily living^22^, wrist movements, and individual digit gestures. These motions are depicted in Fig. 1b which included: index flexion (IF), key pinch (KP), pulp pinch (PP), index point (IP), cylindrical wrap (CW), cylindrical wrap wrist rotate (CR), tripod pinch (TP), wrist extension (WE), wrist flexion (WF), and wrist rotation (WR).

### Data processing

As participants attempted the series of missing hand motions, the sEMG electrodes recorded the electrical activity and data were transmitted to the Delsys System where they were reconstructed, band-pass filtered from 20-450Hz^23,24^, and output as an analog signal. This analog signal was then read by a National Instruments USB 6210 data acquisition system through MATLAB (R2022a, MathWorks, Inc, USA) sampling at 6 kHz. After all data were collected, formatting and feature extraction were performed offline. Movement contraction data were isolated, with 15% cropped from both the beginning and end of each contraction to capture the static muscle state. Notably, the relaxed rest phase contained more data because each movement repetition was followed by an associated rest phase; however, to maintain equal data across all movements, only the rest phase from index flexion was used. The 4-s rest phases were trimmed to 3 s to match the movement data and then processed similarly to ensure equal data amounts. While this approach may have introduced discrepancies, our previous study using the same dataset found that during all attempted missing hand movements, the rest phase exhibited minimal muscle excitation compared to the attempted movements^4^. After isolating all the relevant data, it was concatenated for each missing hand movement to create a single data set which consisted of all 10 repetitions. Then sEMG data were segmented (section “Data segmentation”), features were extracted (section “Feature extraction”), and classifiers were trained (section “Pattern classification”) to evaluate optimal feature sets (section “Feature evaluation”). Evaluation was done with a custom MATLAB script that called functions in BioPatRec software^25^.

#### Data segmentation

Data segmentation specifies a window and time increment in which sEMG data is separated for feature extraction and classification. The typical range for window lengths is 100 to 300 ms^6,26^ and cannot exceed 300 ms as this threshold is perceived as a noticeable control delay if present in real-time prosthetic control applications^27^. However, in an attempt to minimize the time-intensive nature of the feature selection methodology (further described in section “Individual domains and combined domains”), a window length of 300 ms and a time increment of 150 ms were employed. Although this choice would not be realistic for real-time control applications due to latency concerns, it does not compromise the validity of our results as supported by literature suggesting that window lengths between 150 and 550 ms provide no significant difference in classification accuracy^26^.

#### Feature extraction

Feature extraction is the process of identifying relevant sEMG signal characteristics (features) to be classified^28^, which consists of discretized sEMG signals obtained from the time domain, frequency domain, or time–frequency domain. Here we utilized the features provided in BioPatRec and added additional ones to the software package after an extensive search of the literature. These included in total: 31 time domain features, 9 frequency domain features, and 9 time–frequency domain features, a summary of which are provided in Table 2.

**Table 2:** Extracted features from the time domain, frequency domain, and time–frequency domain.

Time domain			
**ID**		**Feature**	**Citation**
1	tmabs	Mean absolute value	^6,8,48,9,16,25,29–31,34,36^
2	tstd	Standard deviation	^6,25,48^
3	tvar	Variance	^6,8,9,25,42,49,50^
4	twl	Waveform length	^6,8,48,50,51,9,16,25,29–31,34,36^
5	trms	Root mean square	^8,9,25,30,31,36,51,52^
6	tzc	Zero-crossing	^6,9,49,50,16,25,29–31,34,36,48^
7	tpks	Number of peaks over the root mean square	^6,25^
8	tmpks	Mean peaks	^6,25^
9	tmvel	Mean velocity	^6,25^
10	tslpch	Slope changes	^6,9,16,25,29,31,34,36,48,50^
11	tpwr	Power	^25^
12	tdam	Difference absolute mean value	^6,25^
13	tmfl	Maximum fractal length	^6,8,25,51,53^
14	tfd	Fractal dimension	^6,25^
15	tfdh	Fractal dimension Higuchi	^6,8,25,53^
16	tren	Rough entropy	^25^
17	tcr	Correlation coefficient	^6,25^
18	tcv	Co-variance	^25^
19	tcard	Cardinality	^25^
20	tHmob	Hjorth mobility	^6,42^
21	tHcom	Hjorth complexity	^6,42^
22	tskw	Skewness (3^rd^ moment)	^6^
23	tdasdv	Difference absolute standard deviation value	^6,9^
24	tkurt	Kurtosis (4^th^ moment)	^6^
25	twam	Willison amplitude: threshold = 0.01	^6,9,49,50,54^
26	tmcer	Multi-channel energy ratio	^6,55^
27	tperc75	75^th^ Percentile	^6^
28	tiabs	Integrated absolute value	^6,9,49,50^
29	thist	Histogram: min max voltage with 9 bins	^6,9,49,50^
30	tssi	Simple square integral	^9^
31	tlogd	Log detector	^9,54^
**Frequency domain**			
1	fwl	Wavelength	^6^
2	fmn	Mean	^6,36,50^
3	fmd	Median	^6,36^
4	fpmn	Peak mean above the root mean square	^6^
5	fpmd	Peak median above the root mean square	^6^
6	fpstd	Peak standard deviation above the root mean square	^6^
7	fmxp	Max peak	^6^
8	fr	Frequency ratio: 20–250 Hz & 251–450 Hz	^6^
9	fe	Frequency energy: 10 Hz bins	^6,52^
**Time–frequency domain**			
1	tfstd	Standard deviation – 4^th^ level wavelet coefficients (DWT)	^6,56^
2	tfvar	Variance – 4^th^ level wavelet coefficients (DWT)	^6,56^
3	tfwl	Waveform length – 4^th^ level wavelet coefficients (DWT)	^6^
4	tfe	Energy – 4^th^ level wavelet coefficients (DWT)	^48,50^
5	tfmxabs1	Maximum absolute value – 4^th^ level wavelet coefficients (DWT)	^6,57^
6	tfmxabs2	Maximum absolute value – tfmxabs1 & all detail levels (DWT)	^57^
7	tfzc	Zero crossing – 4^th^ level wavelet coefficients (DWT)	^50^
8	tfmn	Mean – 4^th^ level wavelet coefficients (DWT)	^6^
9	tfmabs	Mean absolute value – 4^th^ level wavelet coefficients (DWT)	^6^

*DWT* Discrete 4th order Coiflet Wavelet Transform, with 4th level decomposition.

#### Pattern classification

Classification algorithms use sEMG features to identify patterns across the multiple sEMG electrode channels and predict the corresponding movement intent. After a review of the literature, five classifiers were selected based on their classification accuracy and typical training and testing times and are described as follows.

Linear Discriminant Analysis (LDA) is a common technique used to decode motor intent from muscle activity^6,10,16,25,29–34^ in which the variance within the movements feature space is minimized while the difference between the mean of movements is maximized, creating linear boundaries between each movements feature space data. LDA was selected due to its simplicity of implementation, computational demands, and ease of training^28^. We updated LDA in BioPatRec using MATLAB’s *fitcdiscr* function with the discriminant type set to linear.

K-Nearest Neighbor (KNN) calculates the distance from a testing point to its k closest neighbors of the training data to predict the group a movement belongs to. This established method is another common classifier employed to decode hand movements from muscle activity^6,24,29,34^. We implemented KNN in BioPatRec with MATLAB’s *fitcknn* function. Here the distance type was set to Euclidean with k = 1 and the feature data were normalized with the norm-log in BioPatRec^25^. A k = 1 was chosen, as lower values have been shown to improve classification accuracy^6,29,35^. However, it is important to acknowledge that selecting a low k value may limit the generalizability in real-time control applications.

Regulatory Feedback Network (RFN) is a classifier built into BioPatRec that utilizes a connectivity matrix or weights constructed from the average of all training feature vectors with predictions produced through outputs of a negative feedback system^25^. The training type was set to mean with the feature normalization set to unitary range^29^.

Support Vector Machine (SVM) is another commonly implemented classifier used to predict upper limb motor intent from muscle activity^6,24,29–31,33,34,36^. We implemented SVM in BioPatRec with MATLAB’s *fitcecoc* function. Here kernels are used to map data onto separable hyper-planes for classification^6,29^. The SVM had the kernel set to the radial basis function with a scale of 5.9, selected after empirical investigation, and a box constraint of 1. In BioPatRec, the SVM feature normalization range was set between -1 to 1, corresponding to the 0-midrange with 2-range setting^29^.

Decision Tree (DT) has been studied in the context of decoding hand movements from muscle activity^6,29,34^ and we implemented this classifier into BioPatRec with MATLAB’s *fitctree* function. The decision tree uses predictors with greater than or less than criteria to transverse different branches of the tree and make a prediction. The maximum number of splits was set to 100 after empirical investigation and the split criterion was set to Gini’s diversity index.

## Feature evaluation

**Fig. 2:**
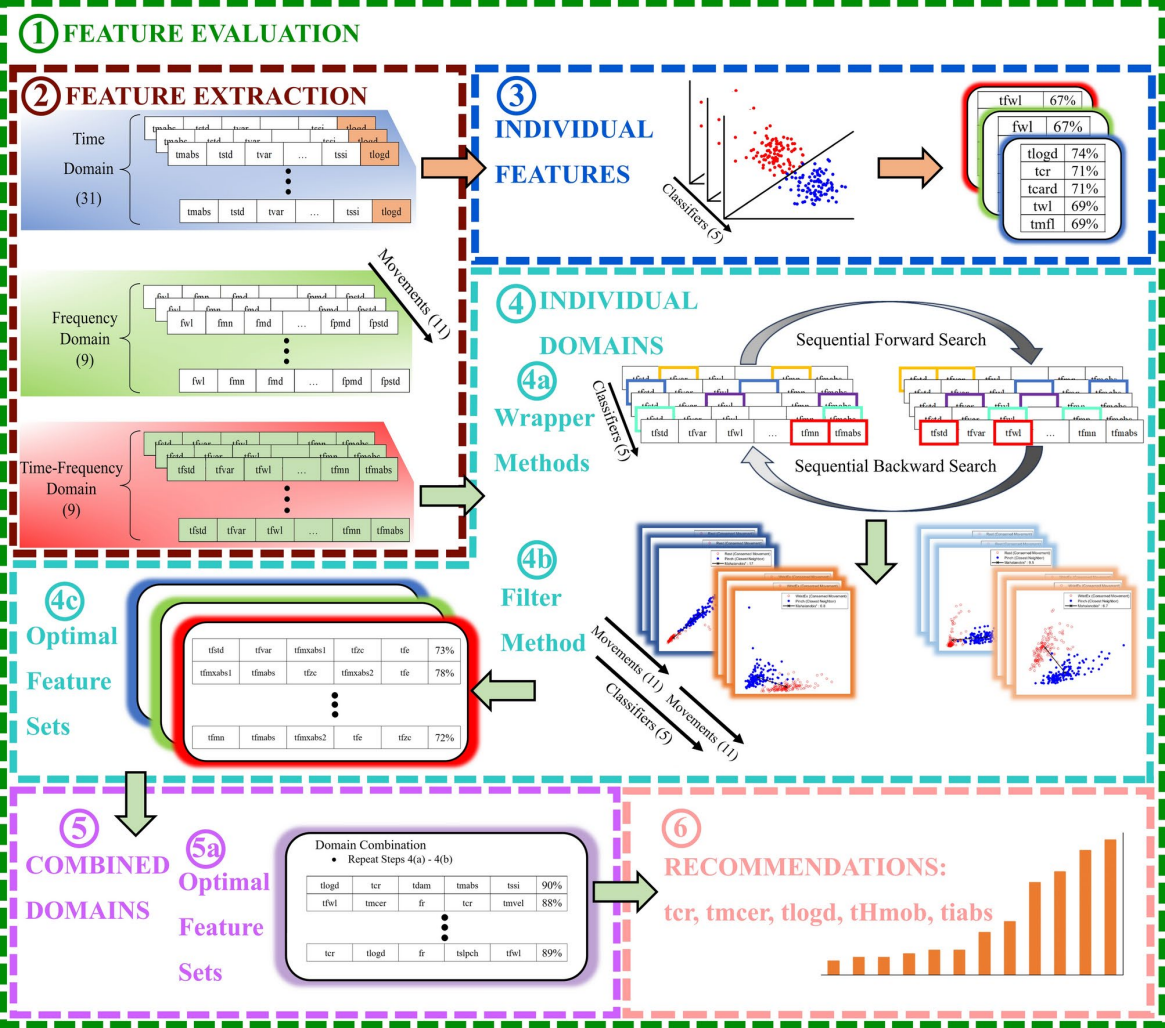
(**1**) Feature evaluation flow diagram. (**2**) Features were extracted for the time domain, frequency domain, and time–frequency domain. (**3**) Individual features were then evaluated across classifiers and the top five features were highlighted. (**4**) Feature sets within individual domains were evaluated via the two wrapper methods (**4a**) followed by the filter method (**4b**) to select the optimal feature set (**4c**). (**5**) The optimal feature sets produced from the individual domains were then combined and (**4ab**) were repeated to produce the optimal combined feature set (**5a**). (**6**) Recommendations for a generalized congenital feature set were then made.

To understand which features and classifier combinations provided the most effective classification performance in predicting attempted hand motions for children with UCBED, a detailed evaluation was performed. Data were collected, features were extracted, and evaluation was performed for each of the five classifiers for the following cases: individual features (49 in total) described in section “Individual features”, individual domains (time, frequency, and time–frequency), and combined domains, both described in section “Individual domains and combined domains”. During evaluation, the classification accuracy for each classifier was obtained by a random split 60–40 cross-validation, where 60% of the data for each movement was used for training and 40% for testing. This process was repeated 100 times, where each iteration randomized the 60–40 training and testing datasets, and the results were averaged^25^. After the feature evaluation was performed, recommendations for a generalized congenital feature set (CFS) were provided (section “Generalized congenital feature set”). Data flow for the feature evaluation can be seen in Fig. 2.

### Individual features

As an essential first step to help us ascertain the potential of decoding important motor information characterized by individual features, we trained and tested five classifiers. The individual features were split into three sets, one for each domain: time domain, frequency domain, and time–frequency domain. The overall classification accuracy for each feature within the specified domain was determined through a 60–40 cross-validation, as previously described. In this procedure, the training and testing data were subject to 100 randomizations each time producing new movement classification accuracies. The classification accuracies from these randomizations were averaged together, and the correct individual movement accuracies were then averaged to produce the overall classification accuracy. A count of the top five highest performing individual features for each classifier and participant was then obtained (shown in Fig. 2 panel 3).

### Individual domains and combined domains

We evaluated sets of features for the individual and combined domains for each classifier in order to understand which sets of features may produce the most optimal classification accuracy. The same methodology was used to evaluate individual and combined domains as shown in Fig. 2 panels 4 and 5, respectively. The only difference between the two was that feature sets for individual domains were evaluated first to find their optimal sets. Upon evaluation, these optimal sets for the individual domains were combined and evaluated to produce the optimal combined-domain feature set. All feature evaluation was done on an individual participant basis.

We identified top-performing feature sets within the individual and combined domains using two wrapper methods (i.e., feature selection algorithms), namely sequential forward search and sequential backward search, as depicted in Fig. 2 panel 4a^7,37^. The classification accuracies were produced as follows: the classification accuracy of every movement was computed for the 100 randomized datasets; then those 100 datasets were averaged together; and finally, the average across all the movements from that averaged dataset was determined. The sequential forward search method loops through all the features and selects the one that produces the average highest classification accuracy. It then loops through the remaining features, each time combining it with the previously chosen feature, ultimately selecting the two features that produce the highest accuracy. This process is then repeated until all the features have been selected, thereby ordering the features based on their contribution to the prediction accuracy. Inversely, the sequential backward search starts with all the features and removes one at a time. Then, the feature that produces the highest classification accuracy when removed is discarded from the total feature set. This process is repeated with the remaining features until only one is left. The feature evaluation process was computationally intensive, requiring approximately six days to complete each participant’s dataset. This was due to the 100 randomizations at each step of the sequential forward and backward search algorithms, repeated across all classifiers and feature domains. After these methods were completed, two datasets were produced: one for sequential forward search and one for sequential backward search. Then for any given classifier and domain, we extracted the feature set containing the top five features produced from each of the two search methods for further analysis.

It should be noted that the two feature sets produced from the forward and backward wrapper methods were not necessarily identical and required a filter method to determine the optimal feature set as depicted in Fig. 2 panels 4b and 4c. Here, we employed the Mahalanobis distance, a typical feature space analysis method that provides a measure for the separability of attempted hand movements i.e., the separability index (SI)^38–41^. This process involved calculating the feature space Mahalanobis distance from one movement to all the remaining movements. The minimum distance among these was then tabulated. This procedure was repeated for each movement, and the average was taken as the SI. This was used as a measure of robustness for the feature sets. A larger SI indicates increased spatial distinction between motions while a smaller SI indicates a decreased spatial distinction^38^. In this way, the optimal set between the search methods was chosen as the one with a larger SI.

The modified SI was defined as the average minimum one-half Mahalanobis distance from the centroid μ_j_ of the jth class to the centroid μ_i_ of the remaining *i* classes, where S_j_ is the covariance matrix of jth class^38^. The inverse of the covariance matrix S_j_ was obtained with the Moore–Penrose pseudoinverse to ensure its existence. This was done because some features were linear combinations of others, and threshold methods such as the Willison amplitude (*twam*) were defined with a single value for all participants, which could have caused sparse feature vectors. Taken together, linear combinations and sparse feature vectors could create noninvertible matrices, which could be addressed by using the pseudoinverse and calculating the magnitude. The modified SI is therefore defined by Eq. (1).

1 $$SI= \frac{1}{11}{\sum }_{j=1}^{11}\left(\underset{i=1,\dots j-1,j+1,\dots 11}{\text{min}}\frac{1}{2}\sqrt{{\left({\mu }_{j}-{\mu }_{i}\right)}^{T}{S}_{j}^{-1}\left({\mu }_{j}-{\mu }_{i}\right)}\right)$$

### Generalized congenital feature set

After the feature sets were produced for the individual domains, they were combined and reevaluated to produce an optimal feature set from the combined domains (Fig. 2 panel 5). The optimal feature sets across classifiers and participants were aggregated to make a recommendation based on a count of the unique number of times each feature occurred (Fig. 2 panel 6). The top five unique features that occurred most often were taken as the generalized congenital feature set (CFS): correlation coefficient (*tcr*), multi-channel energy ratio (*tmcer*), log detector (*tlogd*), Hjorth mobility parameter (*tHmob*), and integrated absolute value (*tiabs*). The calculation methods for these features are detailed by Abbaspour et al. and Phinyomark et al.^6,9^.

## Analysis

### Feature set comparisons

To determine which feature sets may be most suitable for children with UCBED, a comparison across domains and feature sets was performed on an individual participant basis to produce a unique feature set for each domain and classifier. The unique feature sets for each domain are referred to as the following: time domain feature set (TMS), frequency domain feature set (FQS), time–frequency domain feature set (TFS), and combined domain feature set (CDS). Additionally, generalized feature sets described previously in adult literature were also used for comparison. There are a number of proposed feature sets that have been suggested to provide high classification accuracy in able-bodied individuals and adults with acquired limb loss, including ‘the Efficient Feature Set’ (EFS)^6^ and ‘the Hudgins Set’ (HDS)^17^. The EFS consists of the following features: waveform length (*twl*), correlation coefficient (*tcr*), and the Hjorth parameters^42^ (i.e., activity/variance (*tvar*), mobility (*tHmob*), and complexity (*tHcom*)^6^). Additionally, HDS, contains the following features: mean absolute value (*tmabs*), waveform length (*twl*), slope sign changes (*tslpch*), zero crossing (*tzc*), and difference absolute mean value (*tdam*)^17^. Finally, our generalized CFS feature set, produced from an aggregate across participants in this work, was used for comparison.

We analyzed differences in prediction accuracy between the seven feature sets for each of the five classifiers using the non-parametric Friedman test^43,44^. Here, the improved Friedman statistic (F_F_) was then used as described by^44^. The null hypothesis, H_0_, was that all feature sets had the same rank (i.e., the average classifier accuracy across movements will be the same regardless of the feature set). We selected a significance level of α = 0.05 and determined the critical value of F(6,60) = 2.25 to evaluate statistical differences. If the improved Friedman statistic was greater than the critical value (F_F_ > 2.25) then the null hypothesis was rejected. When this occurred, we proceeded with pairwise comparisons of the seven feature sets for a given classifier utilizing the post-hoc Nemenyi test^45^. The critical distance value of 2.72 was determined for the two-tailed Nemenyi test at a significance level of α = 0.05, as described by Demšar et al*.*^44^. If the difference in ranked classification accuracy between any pair of feature sets exceeded the critical distance, it was deemed statistically significant.

### Congenital feature set assessment

Our new congenital feature set was isolated in our analyses to further examine its efficacy as a generalized set for children with UCBED. This included comparisons of its accuracies across classifiers to understand which classifier may provide the highest performance (section “Classifier comparisons”). An investigation of training and testing times was also performed to understand the computational expense (section “Computational expense”), an important aspect for future applications of real-time control. Additionally, we employed movement reduction techniques to identify a subset of high-performing missing hand motions (section “Movement reduction”); this is a practical consideration for prosthetic control in which identifying a subset of highly accurate hand movements may be most useful in executing activities of daily living.

#### Classifier comparisons

Classifier comparisons were performed with the Friedman test as previously described in section “Feature set comparisons”. The null hypothesis, H_0_, was that all classifiers have the same rank, that is, the accuracy across movements was the same regardless of the classifier algorithm. We selected a significance level of α = 0.05 and calculated the critical value of F(4,40) = 2.60 to assess statistical differences. As before, if the improved Friedman statistic (F_F_) was greater than the critical value, then the null hypothesis was rejected, namely, that the rank-based classifier accuracies across movements was not the same for all classifiers. Subsequently, we performed pairwise classifier comparisons using the post-hoc two-tailed Nemenyi test at a significance level of α = 0.05. The critical distance value of 1.84, calculated following Demšar et al*.*^44^, was used to determine statistical significance. This significance was defined as the difference in the ranked classification accuracies between any pair of classifiers that exceeded the critical distance.

#### Computational expense

The training and testing times for the generalized CFS were then obtained for each classifier and participant to understand the computational expense. These computational demands were assessed on a Lenovo PC with the following specifications: a 64-bit Windows 11 operating system with 32 GB of RAM and an Intel core i7-8550U at 1.80 GHz (Intel Corp, USA). The first computational demand, training time, is defined to be the duration to train a classifier and tune hyperparameters. The second computational demand is testing time, which is defined as the transitory period for the offline classifier to predict the labels (missing hand movements). The testing time was used as a metric to assess the potential for real-time control since any value exceeding the 300 ms threshold results in diminished prosthetic control^27^.

#### Movement reduction

Movement reduction was performed for each classifier on the CFS feature set. The reduction procedure involved training a classifier with all attempted hand movements and discarding the movement that produced the lowest classification accuracy. The remaining movements were then used to retrain the classifier, and this process was repeated until only two movements were left. In this way, we identified how the classification accuracy increased with a decrease in attempted hand movements. This is significant because individuals using multi-grasp prostheses will typically employ a limited subset of hand movements. Notably, research has shown that 6–9 hand movements can account for nearly 80% of daily activities^22,46^. Therefore, we propose investigating a subset of five movements for children with UCBED and identifying those subsets that surpass the minimum threshold of 85% classification accuracy needed to promote device usability^28^. It is important to note that the rest state was not considered part of the movements to be discarded because it is an essential state for prosthetic control. Consequently, with every reduction, the rest state was always included, even when there were only two movements remaining, i.e., rest and one other movement were present at the end.

## Results

### Individual features

**Fig. 3:**
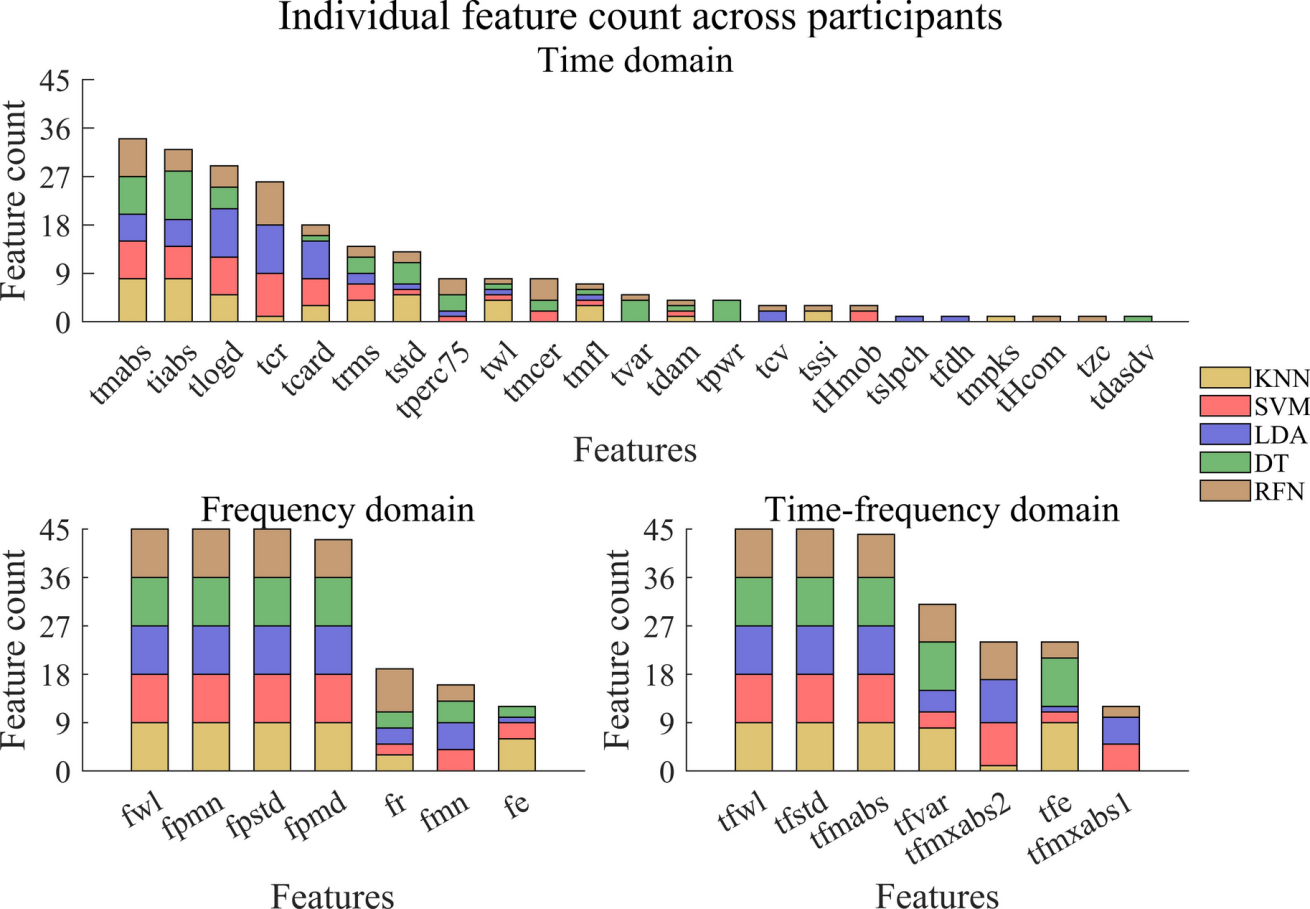
Cumulative count of domain-specific high-performing individual features. The top five high-performing features were identified and counted for a given participant and classifiers. This process was then repeated for every participant, with the results highlighted for the various classification algorithms. The top panel displays the count for the time domain, while the bottom left panel shows the frequency domain, and the bottom right panel shows the time–frequency domain. A count of 45 means that the given feature was in the top five for all nine participants and five classifiers.

We identified and counted the top five performing individual features for a given participant and classifier. This process was performed for each feature domain and repeated for every participant and classification algorithm. The total possible occurrences for one feature across the five classifiers and nine participants was 45. Therefore, a higher count for an individual feature would indicate that it was more often among the top features for each participant (Fig. 3). Here, we highlight four of the top-performing features for the time domain: *tmabs* (34/45), *tiabs* (32/45), *tlogd* (29/45), and *tcr* (26/45). Similarly, the top-performing features for the frequency domain were: *fwl* (45/45), *fpmn* (45/45), *fpstd* (45/45), *fpmd* (43/45). Finally, in the time–frequency domain, the top-performing features included: *tfwl* (45/45), *tfstd* (45/45), *tfmabs* (44/45), and *tfvar* (31/45). It is important to note that when examining classifiers, KNN demonstrated numerically higher classification accuracies for each participant’s top-performing individual features, while RFN exhibited the lowest accuracies. Figure 3 illustrates the cumulative count of domain-specific features for each classifier, obtained by counting the top five high-performing features that occurred for each participant. Detailed tables showcasing the classification accuracies of all individual features for each participant are provided in the Supplementary Tables online.

### Generalized congenital feature set

**Fig. 4:**
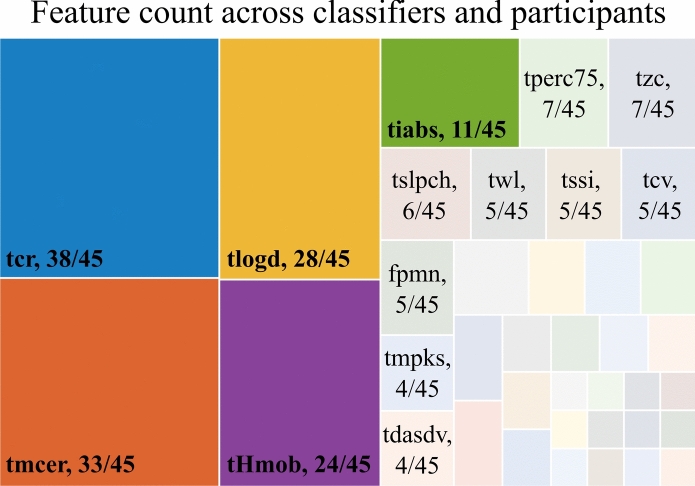
Feature count for recommending the generalized congenital feature set (CFS): correlation coefficient (*tcr*), multi-channel energy ratio (*tmcer*), log detector (*tlogd*), Hjorth mobility parameter (*tHmob*), and integrated absolute value (*tiabs*). The maximum occurrence for an individual feature within the combined domain feature sets was 45, indicating that a single feature could be present in the feature set for all five classifiers and nine participants. The CFS features accounted for 134 occurrences out of the possible 225, representing a total of 60%. In this context, the total possible occurrences result from the presence of the five features within the combined domain set for each of the five classifiers and all nine participants, totaling 225 occurrences.

In the assessment of optimal combined domains, as detailed in section “Individual domains and combined domains”, distinct sets of five features were generated for each participant and classifier. Aggregating these results as displayed in Fig. 4, we identified five features—*tcr*, *tmcer*, *tlogd*, *tHmob*, and *tiabs*—that occurred most frequently, forming the recommended generalized congenital feature set (CFS). Moreover, the CFS accounted for 60% of the total occurrences across participants, classifiers, and features. These results highlight the prevalent features present among the majority of participants, suggesting potential generalizability.

### Feature set comparisons

**Fig. 5:**
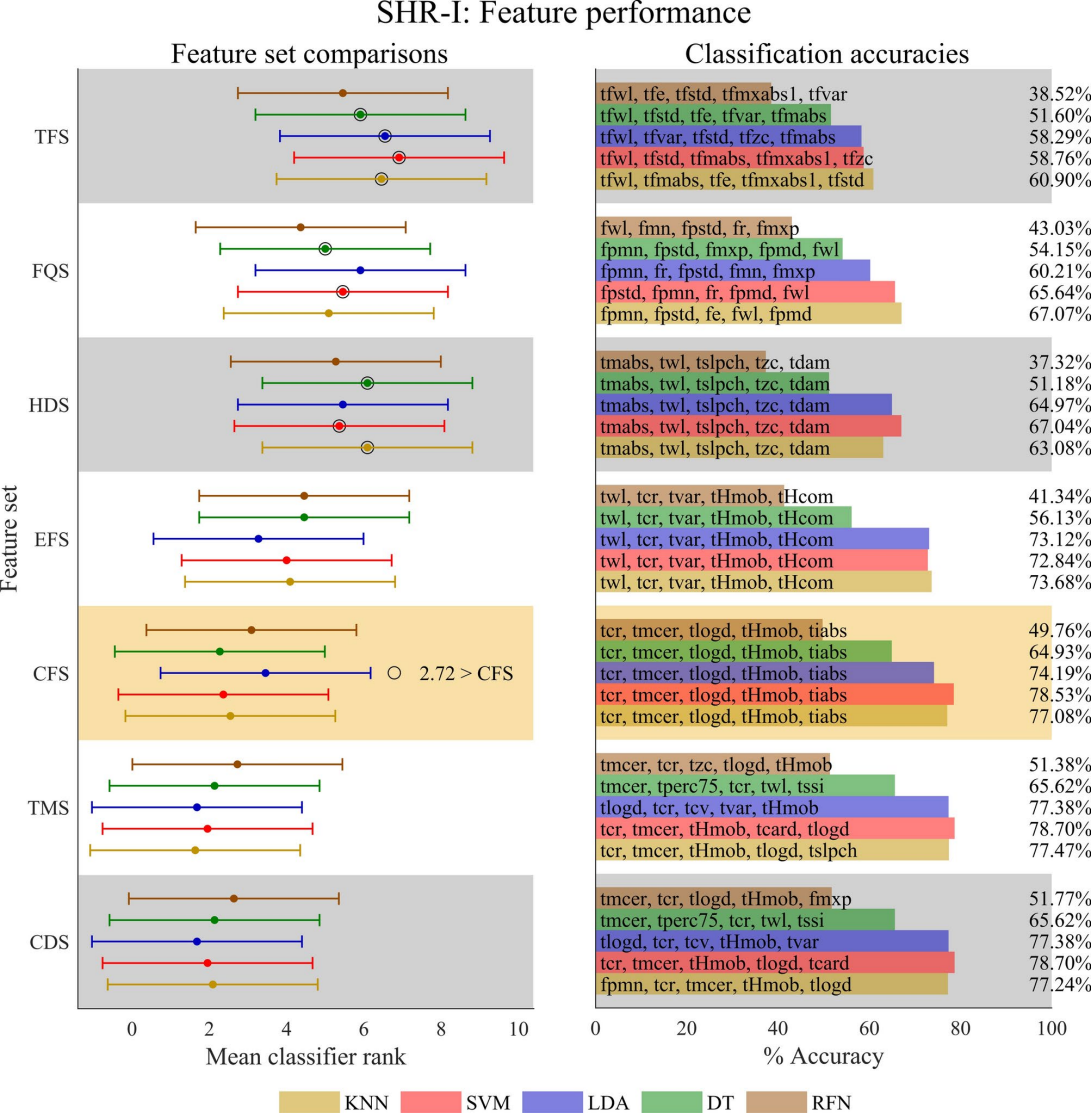
Investigation of feature set classification accuracy for participant SHR-I. The left panel shows the pairwise comparisons for the feature sets on an individual classifier basis. The Friedman test, with a critical value of F(6,60) = 2.25 at α = 0.05, was used to determine if average accuracy ranks significantly differed from the mean rank. Classifier F Statistics (KNN: F_F_ = 45.36, SVM: F_F_ = 51.99, LDA: F_F_ = 56.89, DT: F_F_ = 21.63, RFN: F_F_ = 4.3) confirmed significant differences within each classifier’s feature sets. A post-hoc Nemenyi test with a critical distance of 2.72 at α = 0.05 identified superior feature sets as indicated by pairs outside the critical distance marked by the interval bars. Each classifier is color-coded for easy comparison, with lower average ranks indicating better classification accuracy. Feature sets significantly different from the highlighted congenital feature set (CFS) were marked with an outer black ring. The right panel displays classification accuracies, which range from approximately 39% to 79%, alongside the corresponding feature sets, aligning them with the ranked accuracy shown in the left panel.

To identify the feature sets that have higher classification accuracy for children with UCBED, we performed feature set comparisons with the Friedman test, followed by the post-hoc Nemenyi test. For one participant and one classifier, we performed pairwise comparisons of the seven feature sets, this was then repeated for each of the classifiers, and then for every participant. Statistical significance between any pair of the seven feature sets is discernible when the displacement between the pair exceeds the critical distance of 2.72. As demonstrated in participant SHR-I (Fig. 5), we found higher classification accuracies and few to no statistical differences in the pairwise comparisons of TMS, CDS, CFS, and EFS feature sets for each classifier. Overall, TFS, FQS, and HDS feature sets had lower classification accuracies and exhibited the majority of statistical differences when compared to the remaining feature sets, with few exceptions. The detailed results for each participant can be found in the Supplementary Figures online.

In general, the feature sets decreased in numerical accuracy in the following order: CDS, TMS, CFS, EFS, HDS, FQS, and TFS, as illustrated in Fig. 5 and the Supplementary Figures online. Upon further investigation, we found that the KNN, SVM, and LDA classifiers had numerically higher accuracies, while RFN had the lowest, followed by DT (see Fig. 5 and the Supplementary Figures online). Here, we highlight classification accuracies for the optimal combined domain feature set (CDS) which ranged from 63.87% – 95.37%, 62.61% – 92.86%, 57.33% – 92.87%, 50.79% – 83.19%, and 38.46% – 79.62% for KNN, SVM, LDA, DT, and RFN, respectively. Participant SHR-F had the highest CDS feature set classification accuracy for all the classifiers. Participant SHR-B had the lowest values for LDA and RFN, while SHR-D had the lowest values for KNN, SVM, and DT. In this context, it’s important to highlight that the chance accuracy for decoding the 11 movements is approximately 9%, and it’s noteworthy that all accuracies recorded were above this threshold. Collectively, these results indicate that feature sets, in combination with key classifiers, can be tuned and generalized for children with UCBED to provide higher classification accuracy.

### Congenital feature set assessment

We further investigated the performance of the children-specific CFS feature set which included the comparison of accuracies across classifiers (section “Classifier comparisons”), the evaluation of computational expense (section “Computational expense”), and how classification accuracy improves as we remove the lowest-performing hand movements (section “Movement reduction”).

#### Classifier comparisons

**Fig. 6:**
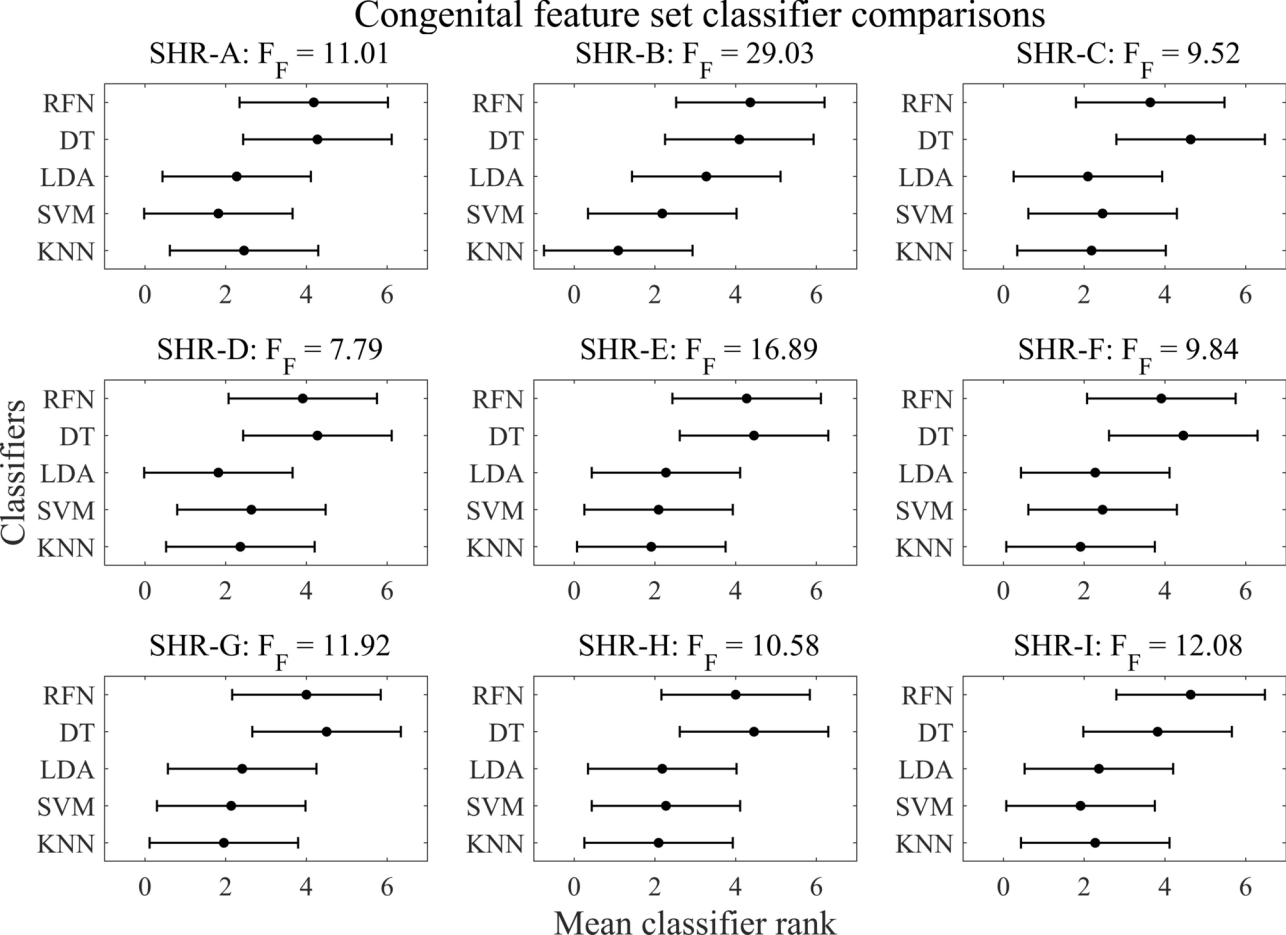
Congenital feature set classifier comparisons. The Friedman test, with a critical value of F(4,40) = 2.60 at α = 0.05, indicated all participants rejected the null hypothesis (F_F_ > 2.60) in favor of the alternative, suggesting a difference across average rank-based classifier accuracies. Here, a lower average rank indicates superior classifier accuracy. Pairwise comparisons were performed using the post-hoc Nemenyi test, with a critical distance of 1.84 at α = 0.05. Statistical significance for a specific classifier is denoted by classifiers positioned outside the critical distance, as indicated by the interval bars.

All pairwise comparisons between SVM, LDA, and KNN classifiers showed no statistical differences in the average ranked classification accuracies and demonstrated consistently high classification (with the exception of participant SHR-B). In contrast, DT and RFN classifiers exhibited both lower classification accuracy and all other observed statistical differences when compared to KNN, SVM, and LDA. The range of classification accuracies for the CFS feature set were as follows: KNN (62.17% – 94.17%), SVM (62.01% – 93.11%), LDA (56.22% – 92.80%), DT (50.65% – 82.43%), and RFN (37.07% – 79.74%). Participant SHR-F exhibited the highest classification accuracy for each classifier, while SHR-B and SHR-D had the lowest. Friedman test statistics and a visual depiction of the post-hoc Nemenyi test are provided in Fig. 6 for all participants. In these graphical depictions, pairwise comparisons between classifiers within the CFS are observed, and statistical differences were determined by those that exceeded the critical distance of 1.84. A lower rank denotes superior classifier accuracy while a higher rank denotes diminished accuracy. The results from pairwise classifier comparisons within the CFS suggest that KNN, SVM, and LDA classifiers provide a significant improvement over DT and RFN in their current states.

#### Computational expense

To understand the associated computational expense for the CFS feature set, we obtained the average training and testing times for each participant and classifier algorithm. On average, we see the RFN classifier had the largest training times ranging from 421 ms – 758 ms, followed by SVM with 560 ms – 637 ms. LDA, KNN, and DT had relatively similar training times, all falling within the LDA range of 243 ms – 362 ms. Additionally, all testing times fell under the 300 ms threshold for usable real-time control^27^, and the range of time values are provided are follows: SVM (18.61 – 19.45 ms), KNN (3.59 – 3.93 ms), DT (2.03 ms – 2.38 ms), RFN (0.32 ms – 0.44 ms), and LDA (0.12 ms – 0.15 ms). Training and testing times for the CFS of each participant and classifier are shown in Table 3. The low training and testing time results suggest effective employment of the CFS across classifiers for future real-time control.

**Table 3:** Computational expense of the congenital feature set (CFS) across classifiers.

Participants	Training time (ms)					Testing time (ms)				
	**LDA**	**KNN**	**RFN**	**SVM**	**DT**	**LDA**	**KNN**	**RFN**	**SVM**	**DT**
SHR-A	276	258	421	589	249	0.12	3.65	0.32	18.61	2.38
SHR-B	290	329	752	574	279	0.12	3.84	0.44	18.94	2.28
SHR-C	243	300	644	592	306	0.12	3.59	0.37	19.45	2.08
SHR-D	362	333	758	605	352	0.12	3.89	0.42	19.18	2.35
SHR-E	349	317	640	564	280	0.12	3.73	0.41	18.80	2.14
SHR-F	345	310	587	560	351	0.13	3.92	0.37	18.66	2.21
SHR-G	323	296	560	591	305	0.13	3.65	0.36	18.76	2.19
SHR-H	349	315	657	599	318	0.15	3.91	0.44	18.85	2.03
SHR-I	344	321	661	637	335	0.12	3.93	0.43	19.25	2.31

#### Movement reduction

We eliminated attempted hand movements one at a time, based on the lowest classification accuracy; to examine the relationship between the number of movements and classification accuracy. Although we reduced the number of movements to two (rest and one other motion), our point of interest was a reduced state of five (rest state and four other motions). This was done primarily because prosthesis wearers generally use a smaller selection of hand movements to assist in activities of daily living. The other point of interest was a minimum 85% classification accuracy threshold, which is needed to mitigate wearer frustration and promote device usability^28^.

**Fig. 7:**
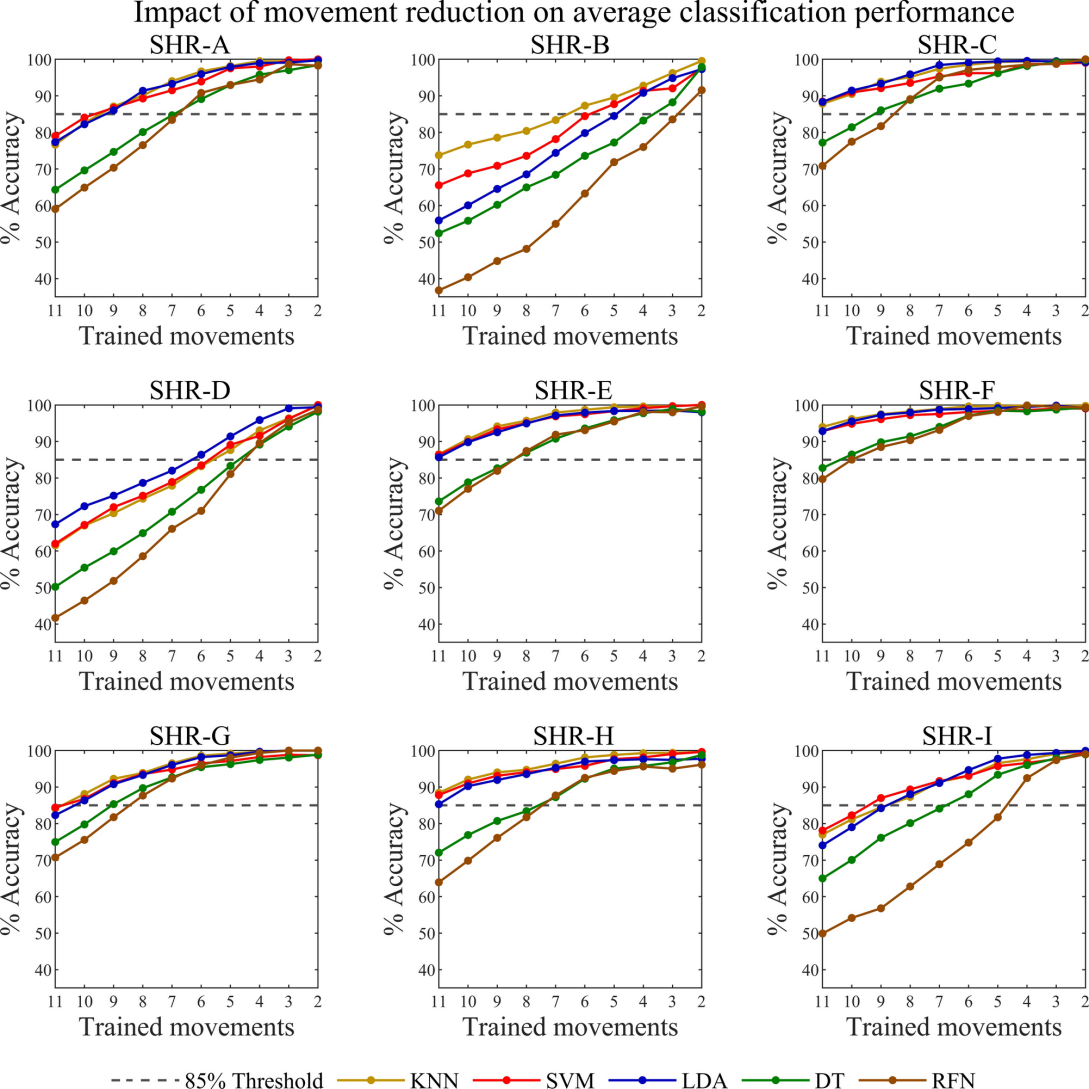
Impact of movement reduction. Movements were reduced from all 11 to a set of 2, as annotated on the line plots. Note that the rest state was not removed during this reduction process. The movement with the lowest classification accuracy was omitted, and the remaining movements were used to retain the given classifier. This process was repeated until only two movements remained: rest and one other. The dashed line indicates the suggested usability threshold for device control, set at 85% accuracy^28^.

The majority of participants across classifiers had accuracies greater than the 85% threshold for the reduced set of five movements. For the LDA classifier, participants had accuracies ranging from 91.38% – 99.45% except for SHR-B, who had a classification accuracy of 84.49% (less than the 85% threshold). All participants had KNN classification accuracies greater than the threshold, ranging from 87.64% – 99.74%. With the exceptions of SHR-B (71.86%), SHR-D (81.08%), and SHR-I (81.72%), all other participants had RFN classification accuracies greater than 85%, ranging from 93.00% – 98.09%. For the SVM classifier, all accuracies were above the threshold and ranged from 87.73% – 98.38%. Finally, for the DT classifier, SHR-B and SHR-D had classification accuracies of 77.23% and 83.31%, respectively, while all other participants ranged from 92.95% – 98.45%. Movement reduction plots for all participants are provided in Fig. 7.

**Fig. 8:**
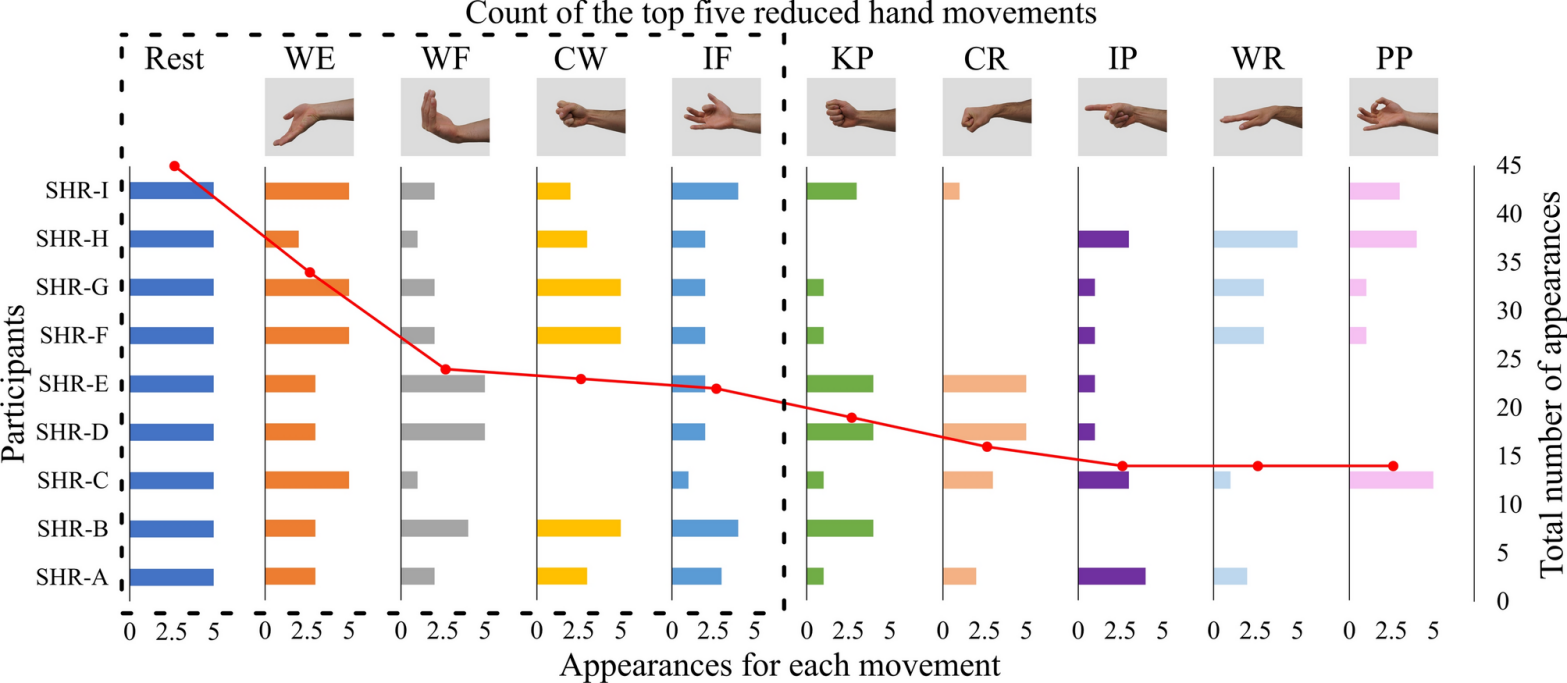
Count of the top five reduced hand movements. In a given column, the individual participant’s numerical bar value indicates the cumulative occurrence of the movement across all five classifiers. Notably, rest was not removed during reduction and thus occurs in every participant and classifier. The superimposed red scatter plot illustrates the overall occurrence of a specific movement across all nine participants and five classifiers; therefore, the maximum possible occurrence was 45 (as seen on rest).

To identify common movements within the reduced set of five across participants, we first counted the frequency of each movement’s occurrence across the five classifiers for a single participant. Consequently, a single movement could appear in each of the five classifiers. This process was repeated for all nine participants, potentially resulting in a total occurrence of one movement 45 times and for five movements 225 times. The rest state, which was kept fixed and not removed to establish a foundation for predicting motor intent, retained a total occurrence of 45. Figure 8 depicts a count of the reduced set of five movements for each participant and aggregated across all participants. Here, we observed that wrist extension (WE) and wrist flexion (WF) accounted for 34 out of 45 (76%) and 24 out of 45 (53%) of the top single-movement occurrences, respectively. Aggregating rest, WE, WF, CW, and IF accounted for 148 out of 225 total occurrences (66%). These results highlight that some movements may be easier for all participants to envision and attempt.

## Discussion

### Unique features can be identified for children with UCBED

When we analyzed the individual features and feature sets, unique features were identified for children with UCBED. The top-performing individual features identified across classifiers and participants for the time domain were: *tmabs*, *tiabs*, *tlogd*, and *tcr* (Fig. 3). It is interesting to note that three of these top-performing individual features (*tcr*, *tlogd*, and *tiabs*) were also observed frequently within the tuned feature sets for the time and combined domains across every classifier (see Fig. 4, Fig. 5, and the Supplementary Figures online). These three features showed up in the generalized congenital feature set (CFS) which consisted of *tcr*, *tmcer*, *tlogd*, *tHmob*, and *tiabs*.

### Children with UCBED benefit from certain feature sets

Numerous feature sets and classification algorithms have been tuned for adult able-bodied individuals, with the common assumption that they will seamlessly translate to those with acquired amputation^14^. Nevertheless, these assumptions are not rigorously tested in affected populations. Unlike individuals with traumatic amputation, the unique population studied here presents more uncertainty as they were born with limb absence and, consequently, have never actuated an intact hand. Interestingly, the efficient feature set (EFS) developed on able-bodied individuals^6^ performed well for those with congenital limb deficiency. Although not statistically significant relative to the EFS, the newly developed generalized congenital feature set (CFS) provided numerically higher classification accuracies and was comparable to each participant’s tuned time-domain and combined-domain feature sets.

When we analyzed the feature sets for each classifier and participant, very few statistical differences were obtained for the following feature sets: TMS, CDS, EFS, and CFS (see Fig. 5 and the Supplementary Figures online). The majority of pairwise statistical differences that were observed occurred between the previously mentioned feature sets and the TFS, FQS, and HDS sets (which had lower classification accuracies). This suggests that pure frequency, time–frequency, or even the commonly implemented generalized Hudgins set do not provide sufficient information for optimal classification in children with UCBED. Moreover, although the CDS feature set was produced from the optimal set of each domain on an individual participant basis, we found that the majority of features within the CDS sets ended up being from the time domain. Evidently so, these results are in line with previous research that attributes the best performing features to the time domain^23^, which is also the case here for children with UCBED. It is worth noting that the normalization process applied to the features varied across classification algorithms, which may have influenced the observed results. This variability in normalization could have affected the classification accuracies and thereby the statistical differences. The choice of feature normalization is inherently dependent on the classification algorithm used^25^. The different normalization methods were selected for each classifier based on their demonstrated effectiveness in enhancing classification accuracy^25,29^. Given that our study aimed to maximize accuracy, the appropriate selection of feature normalization on a classifier basis was warranted.

Interestingly, participant SHR-F had the highest classification accuracy for the CDS feature set of each classifier (LDA: 92.87%, KNN: 95.37%, RFN: 79.62%, SVM: 92.86%, and DT: 83.19%). It is important to note that this participant exhibited seemingly unlikely factors that would merit good classification accuracy: (1) they only reported the use of a passive device as opposed to a myoelectric prosthesis; (2) they were not the oldest, but rather in the upper middle age range at 16 years old; (3) neither did they have the largest limb length (11.5 cm) or circumference (23.5 cm). Despite these demographics, the only difference between this participant and the others was their sex (female). In contrast, the two lowest accuracies for the CDS feature set of each classifier were attributed to SHR-B (8 years old) and SHR-D (9 years old), the two youngest participants of the cohort. Moreover, the oldest participant SHR-A (20 years old) had the smallest residual limb circumference (15 cm) and had the third lowest scores for LDA, KNN, and DT. It follows that a combined effect of limb size with sex- and age-related cognitive demands may impact the ability to decode motor intent, suggesting that further research in these areas is warranted.

### The congenital feature set is an effective generalized set

The CFS feature set demonstrated generalizability and efficacy in decoding motor intent for our cohort of children with UCBED. Notably, the features that comprise the CFS belong to the time domain, with the subtle exception of the Hjorth mobility feature (tHmob). This feature is derived as the square root of the ratio between the variance of the time domain signal’s first derivative relative to the variance of the original signal^42^. Additionally, the mobility feature can be interpreted in the frequency domain as the standard deviation of the power spectrum^42^. Thus, while all the CFS features were categorized in the time domain, the mobility feature offers a direct interpretation in the frequency domain.

To further assess the practical implications of the CFS feature set, a comparative analysis was conducted to evaluate its classification accuracy against other feature sets. This analysis revealed no statistical differences in classification accuracy when compared to the individually tuned feature sets TMS and CDS, as well as the generalized EFS feature set (with the exception of SHR-B). It is important to note that, in general, the CFS exhibited numerically higher accuracy than the generalized EFS. Furthermore, while tuning feature sets (TMS and CDS) on an individual participant basis has the potential to improve classification accuracy, the observed numerical gains are negligible when contrasted with the generalized CFS. Therefore, we recommend the adoption of the CFS feature set for participants with UCBED.

When revisiting participant SHR-F, we found they also exhibited the highest classification accuracy for each classifier relative to all other participants, while SHR-B and SHR-D displayed the lowest. Moreover, we found that when attempted hand movements were reduced to a set of five (including rest state) the majority of participants across classifiers had average accuracies greater than the 85% threshold (Fig. 7). The participants that did not meet this threshold in totality were nonetheless able to meet it for at least two classifiers (SVM and KNN). For example, participants SHR-B and SHR-D did not meet this threshold for the following values: SHR-B (LDA 84.49%, RFN 71.86%, and DT 77.23%) and SHR-D (RFN 81.08%, DT 83.31%). These results further highlight the need to study the effects of age and sex in decoding motor intent, given that SHR-B and SHR-D were the youngest two participants. Collectively, these findings reveal the efficacy of the generalized CFS feature set considering that all participants were able to perform 5 hand movements above the 85% threshold.

What’s more, we found that within the top five reduced hand movements that were aggregated across participants, the following gross motor movements emerged (Fig. 8): wrist extension (WE), wrist flexion (WF), and cylindrical wrap (CW). This is likely attributed to the fact that participants were born with limb deficiency and conceivably did not fully develop fine motor skills, hence why intricate hand movements may have proved challenging. It is astounding that the only digit movement, index flexion (IF), emerged following the bulk-movement sequence (WE, WF, and CW). This discovery comes into view in light of the fact that index flexion is unlike any other movement that participants were prompted to attempt, despite being a fine motor skill. Perceivably, this muscle activity is uniquely distinguishable from the other movements which can be seen by its presence in the top five reduced hand movements. Finally, since the tripod pinch (TP) did not occur in any of the five reduced movements observed across participants, we inferred that it shares similarities with the pulp pinch, a comparison frequently made by participants during testing. Comprehensively, we can glean that notwithstanding the dominance of gross movements, participants had the capacity to actuate unique motions, which can thereby be improved with regular prosthesis use and proper training.

We found no statistical differences in the average ranked classification accuracies for the CFS feature set when we made pairwise comparisons between SVM, LDA, and KNN classifiers, with the exception of SHR-B (Fig. 6). However, the majority of statistical differences were observed from the previously mentioned classifiers to DT and RFN. Although the DT exhibited lower classification accuracies, in previous work, it has indicated similar performance to the other classifiers^6,29^. This may suggest that the DT classifier requires additional tuning. In general, RFN also produced the lowest classification accuracy across participants, which aligns with other comparisons made to this classifier^25,29^. Additionally, when examining the computational expense of the CFS, we found minimal time delays within classifiers across participants (Table 3). Furthermore, all classifier training times were within a reasonable range of less than 758 ms. Similarly, the testing time was also within a range suitable for future implementation of real-time control, with all values less than 19.45 ms. From these performance results, we deduce that SVM, LDA, and KNN are ideal classifiers for future investigations as currently implemented in MATLAB since they produced high accuracies and relatively low training and testing times.

## Conclusion

To date, we have not found any other studies involving children with UCBED that investigate sEMG classification algorithms and tuned feature sets. Our work suggests three crucial points: (1) unique features arise for these children, (2) certain feature sets are beneficial for optimal classification, and (3) the newly developed generalized congenital feature set (CFS) effectively decodes motor intent. In tandem with these three points, we propose that cognitive demands related to age, sex, and limb size may critically influence motor intent. Further investigation of these factors with larger cohorts is needed to make definitive conclusions. In general, we found that the range of accuracies obtained for the CFS across all movements and classifiers was 73.8% ± 13.8%. However, the use of overlapping windows for feature extraction may have resulted in shared information between training and testing sets, leading to potential information leakage that could have influenced classification accuracy^47^. These results were further impacted by the difficulty children experienced in attempting repeated hand movements to the same degree. Therefore, we suggest that with physical conditioning and training, children may be able to effectively control multiple movements of dexterous upper limb prostheses. Moreover, when the 11 movements were reduced to a subset of 5, we found that all participants were able to reach the ideal threshold (85%) with accuracies of 96.5% ± 6.6%. This is an encouraging discovery since multi-grasp prosthesis wearers generally use only a small subset of movements and reaching the ideal threshold would thereby mitigate wearer frustration and ensure device usability^28^. We have found that the limited number of studies that investigate UCBED cohorts do not adapt tuned classifiers and feature sets for this unique population. Generally, these studies only apply commercially available sEMG control systems or the Hudgins feature set, both of which are tailored to adults with acquired amputations^3,16^. Our work has shown that the Hudgins set often performs statistically worse when compared to the generalized CFS feature set or the individually tuned time-domain and combined-domain feature sets for each participant. Since the CFS shows promising results as a generalized feature set, further endeavors should be undertaken to determine its robustness on both a feature space level and in larger cohorts. In conclusion, the results indicate that children with UCBED have the ability to actuate their muscles in ways that classifier algorithms can decode and use for control of dexterous upper limb prostheses. Ultimately, there is a need to bridge the gap between our offline work performed on pre-recorded datasets and that of real-time control. Bridging this gap would enable us to develop effective devices for the unique clinical population.

## Supplementary Information

Supplementary Information 1.

Supplementary Information 2.

## Data Availability

The data that support the findings of this study are available from Shriners Children’s – Northern California but restrictions apply to the availability of these data, which were used under license for the current study, and so are not publicly available. Data are however available from the corresponding author upon reasonable request and with permission of Shriners Children’s.
