# Supplementary material for: Surface electromyography evaluation for decoding hand motor intent in children with congenital upper limb deficiency
Source: Sci Rep. 2024 Dec 30;14:31741. doi: 10.1038/s41598-024-82519-z (PMC11685410; doi:10.1038/s41598-024-82519-z)
Supplement: Supplementary file 1 — Supplementary Information 1. [file 41598_2024_82519_MOESM1_ESM.pdf]

Surface electromyography evaluation for decoding hand motor intent in children with congenital upper limb deficiency

Marcus A Battraw[1], Justin Fitzgerald[2,3,4], Eden J Winslow[2], Michelle A James[5,6], Anita M Bagley[5,6], Wilsaan M Joiner[3,7], Jonathon S Schofield*[1]

[1]Department of Mechanical and Aerospace Engineering, University of California, Davis, Davis CA, USA

[2]Department of Biomedical Engineering, University of California, Davis, Davis CA, USA

[3]Department of Neurobiology, Physiology and Behavior, University of California, Davis, Davis CA, USA

[4]Clinical and Translational Science Center, University of California Davis Health, Sacramento, CA, USA

[5]Shriners Children's – Northern California, Sacramento CA, USA

[6]Department of Orthopaedic Surgery, University of California Davis Health, Sacramento CA, USA

[7]Department of Neurology, University of California Davis Health, Sacramento, CA, USA

*** Correspondence:**
Jonathon Schofield
jschofield@ucdavis.edu

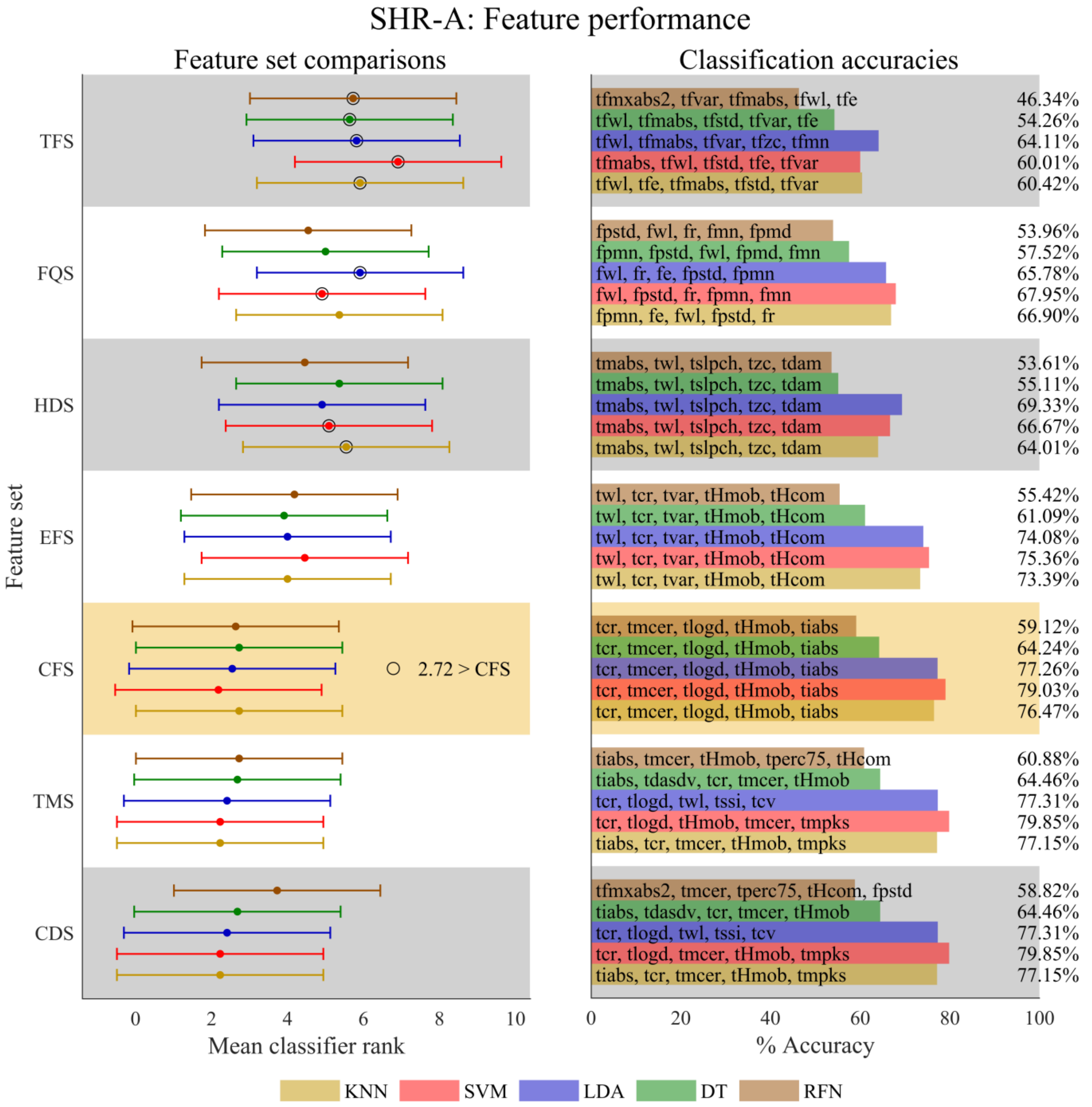

Supplementary Figure S1. Investigation of feature set performance for participant SHR-A. The left panel shows the pairwise comparisons for the feature sets on an individual classifier basis. The Friedman test, with a critical value of F(6,60) = 2.25 at α = 0.05, was used to determine if average accuracy ranks significantly differed from the mean rank. Classifier F Statistics (KNN: $F_F = 12.95$, SVM: $F_F = 26.25$, LDA: $F_F = 11.46$, DT: $F_F = 6.13$, RFN: $F_F = 3.40$) confirmed significant differences within each classifier's feature sets. A post-hoc Nemenyi test with a critical distance of 2.72 at α = 0.05 identified superior feature sets as indicated by pairs outside the critical distance marked by the interval bars. Each classifier is color-coded for easy comparison, with lower average ranks indicating better classification accuracy. Feature sets significantly different from the highlighted congenital feature set (CFS) were marked with an outer black ring. The right panel displays classification accuracies, which range from approximately 46% to 80%, alongside the corresponding feature sets, aligning them with the ranked performance shown in the left panel.

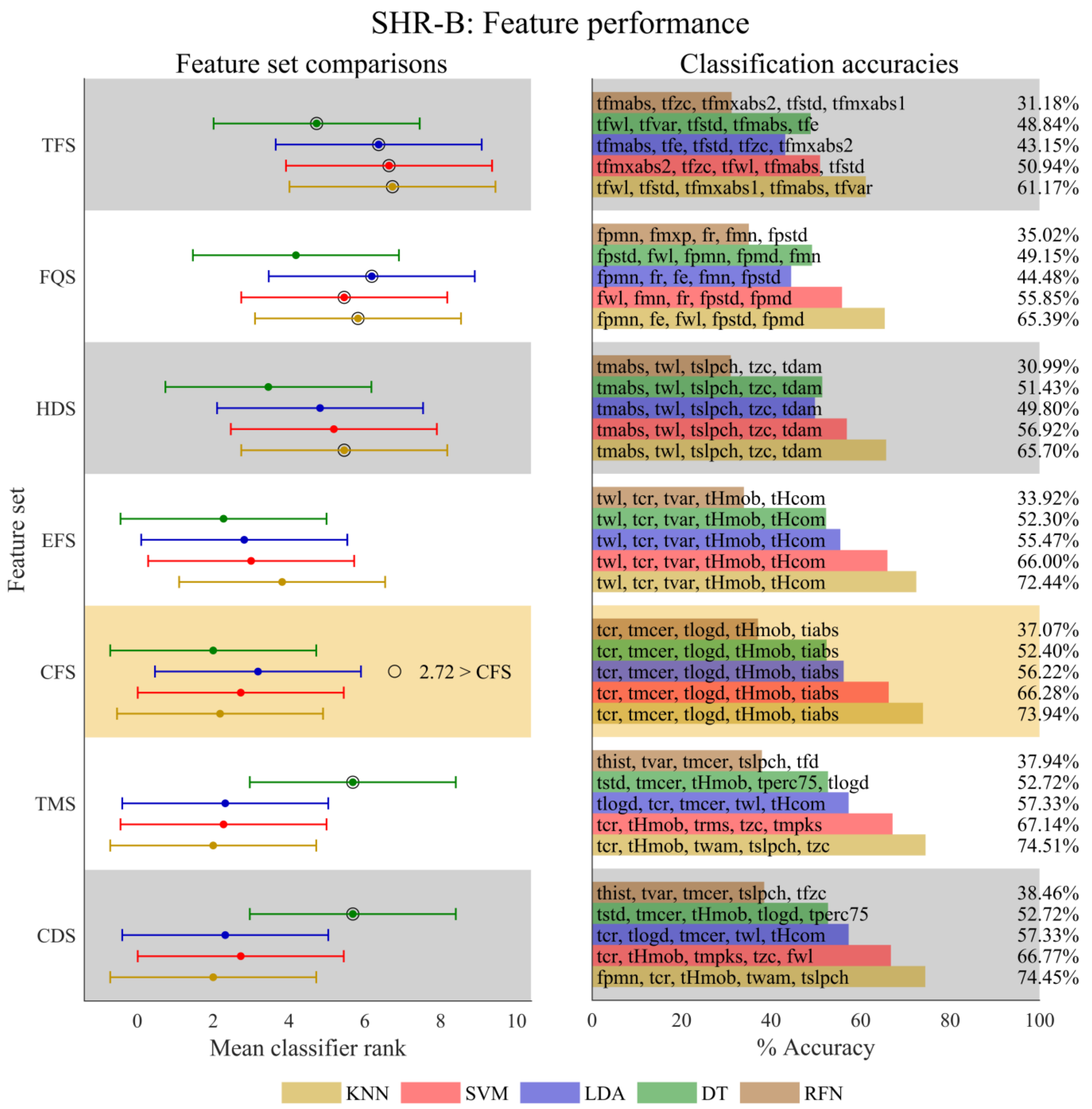

Supplementary Figure S2. Investigation of feature set performance for participant SHR-B. The left panel shows the pairwise comparisons for the feature sets on an individual classifier basis. The Friedman test, with a critical value of F(6,60) = 2.25 at α = 0.05, was used to determine if average accuracy ranks significantly differed from the mean rank. Classifier F Statistics (KNN: $F_F = 63.65$, SVM: $F_F = 17.15$, LDA: $F_F = 20.24$, DT: $F_F = 9.31$, RFN: $F_F = 0.65$) confirmed significant differences within each classifier's feature sets, with the exception of RFN. A post-hoc Nemenyi test with a critical distance of 2.72 at α = 0.05 identified superior feature sets as indicated by pairs outside the critical distance marked by the interval bars. Each classifier is color-coded for easy comparison, with lower average ranks indicating better classification accuracy. Feature sets significantly different from the highlighted congenital feature set (CFS) were marked with an outer black ring. The right panel displays classification accuracies, which range from approximately 31% to 75%, alongside the corresponding feature sets, aligning them with the ranked performance shown in the left panel.

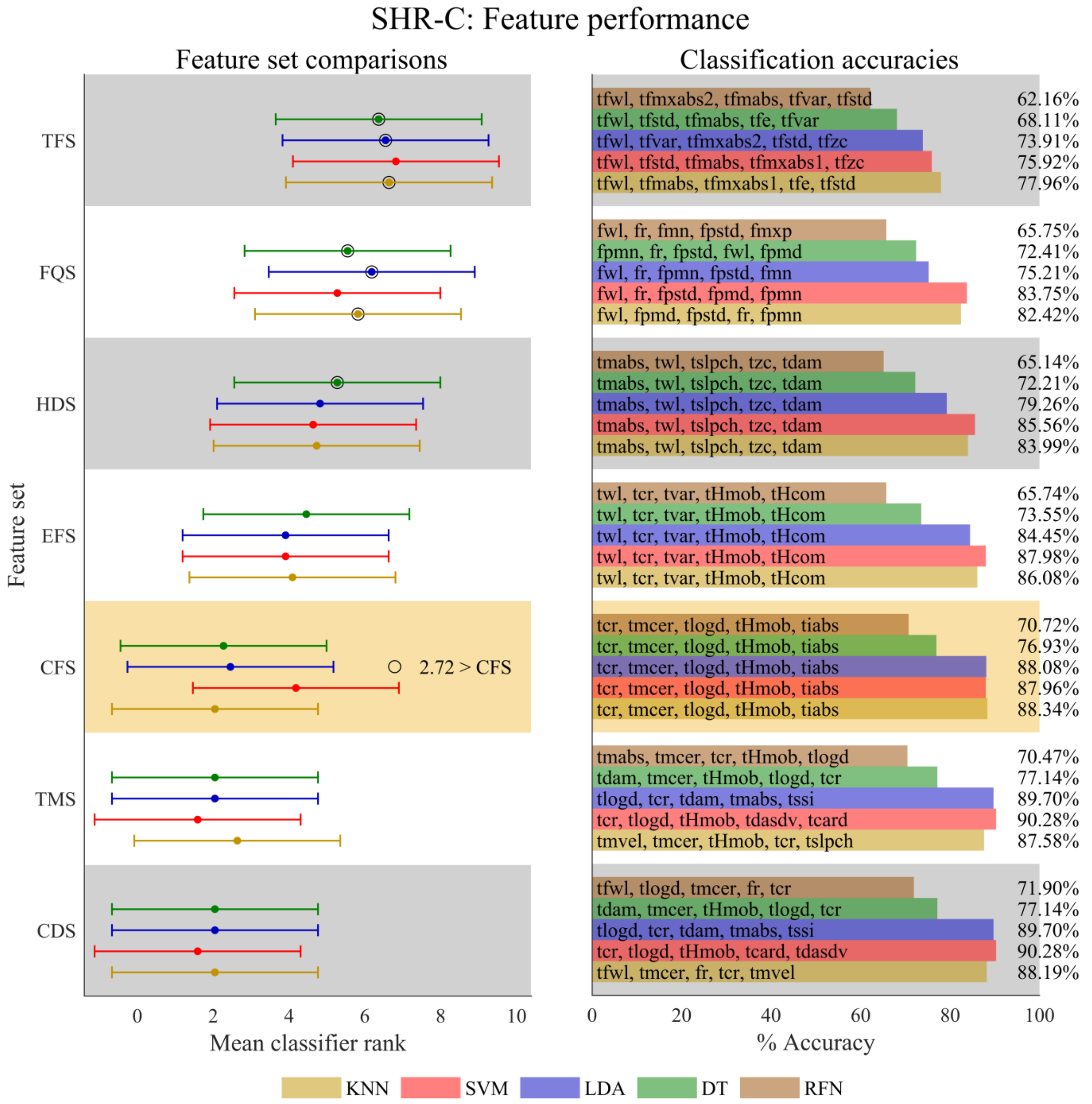

Supplementary Figure S3. Investigation of feature set performance for participant SHR-C. The left panel shows the pairwise comparisons for the feature sets on an individual classifier basis. The Friedman test, with a critical value of F(6,60) = 2.25 at α = 0.05, was used to determine if average accuracy ranks significantly differed from the mean rank. Classifier F Statistics (KNN: $F_F = 26.33$, SVM: $F_F = 33.86$, LDA: $F_F = 36.25$, DT: $F_F = 26.97$, RFN: $F_F = 1.90$) confirmed significant differences within each classifier's feature sets, with the exception of RFN. A post-hoc Nemenyi test with a critical distance of 2.72 at α = 0.05 identified superior feature sets as indicated by pairs outside the critical distance marked by the interval bars. Each classifier is color-coded for easy comparison, with lower average ranks indicating better classification accuracy. Feature sets significantly different from the highlighted congenital feature set (CFS) were marked with an outer black ring. The right panel displays classification accuracies, which range from approximately 62% to 90%, alongside the corresponding feature sets, aligning them with the ranked performance shown in the left panel.

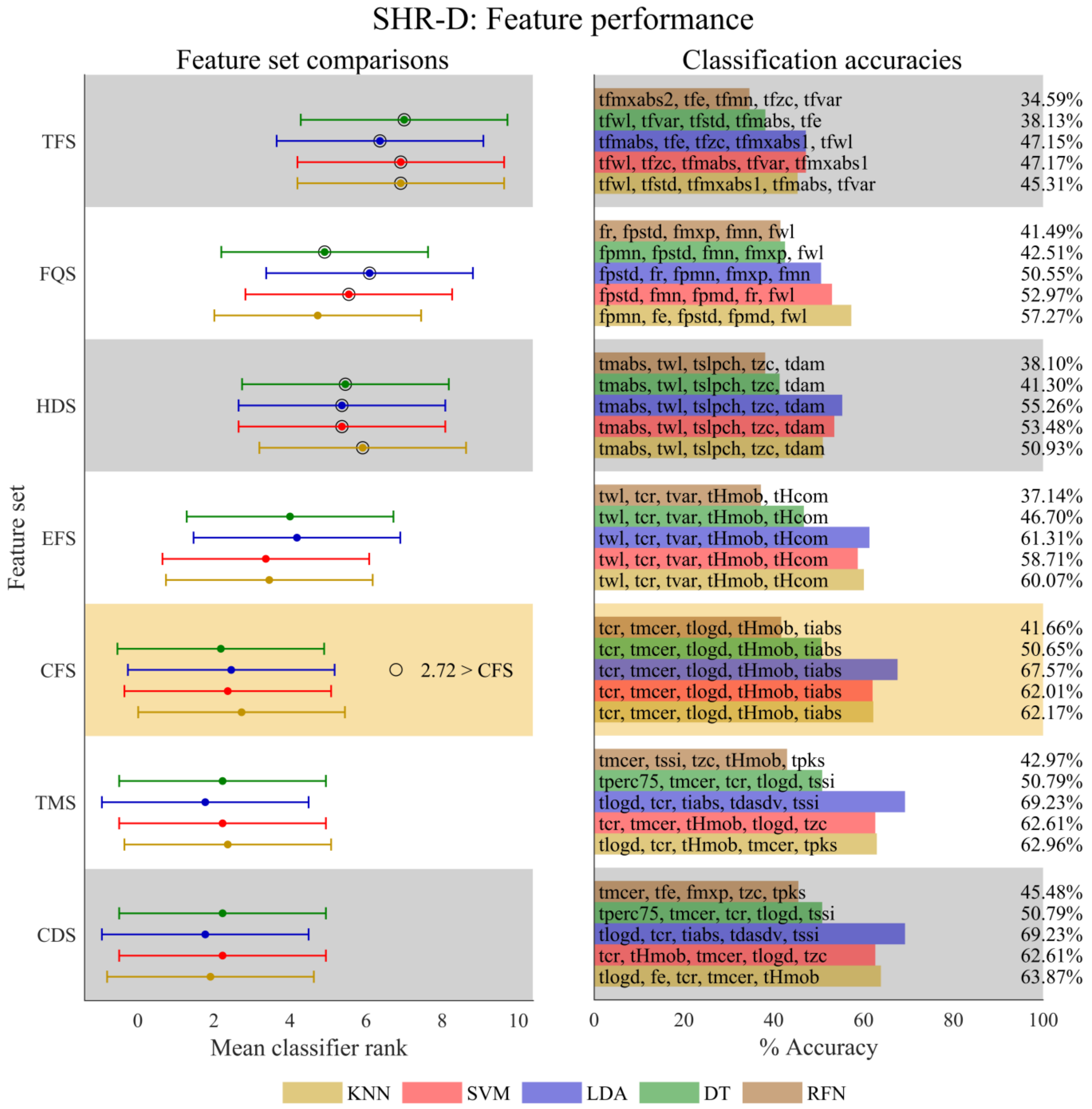

Supplementary Figure S4. Investigation of feature set performance for participant SHR-D. The left panel shows the pairwise comparisons for the feature sets on an individual classifier basis. The Friedman test, with a critical value of F(6,60) = 2.25 at $\alpha = 0.05$, was used to determine if average accuracy ranks significantly differed from the mean rank. Classifier F Statistics (KNN: $F_F = 33.77$, SVM: $F_F = 37.29$, LDA: $F_F = 62.94$, DT: $F_F = 33.30$, RFN: $F_F = 1.20$) confirmed significant differences within each classifier's feature sets, with the exception of RFN. A post-hoc Nemenyi test with a critical distance of 2.72 at $\alpha = 0.05$ identified superior feature sets as indicated by pairs outside the critical distance marked by the interval bars. Each classifier is color-coded for easy comparison, with lower average ranks indicating better classification accuracy. Feature sets significantly different from the highlighted congenital feature set (CFS) were marked with an outer black ring. The right panel displays classification accuracies, which range from approximately 35% to 69%, alongside the corresponding feature sets, aligning them with the ranked performance shown in the left panel.

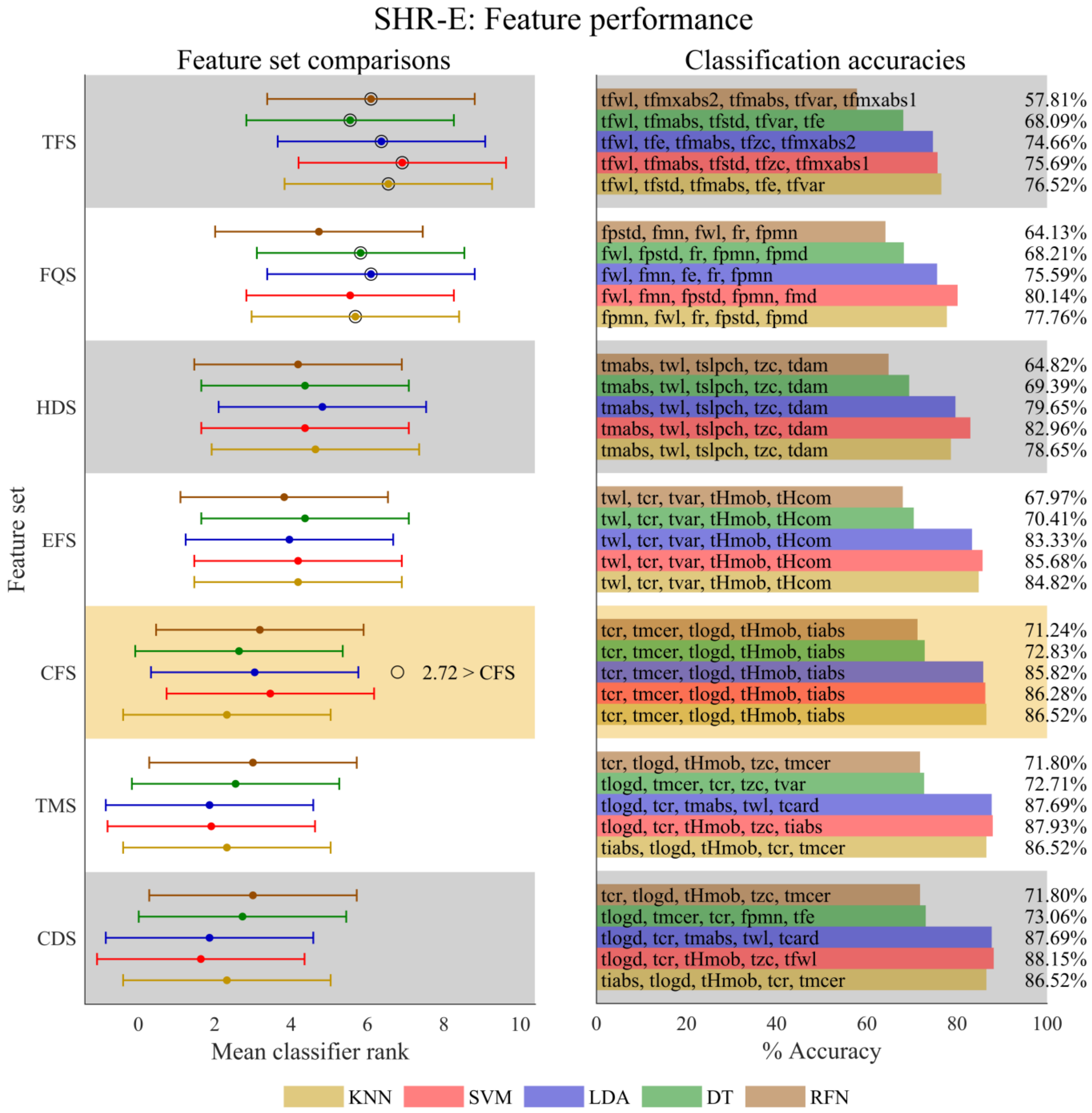

Supplementary Figure S5. Investigation of feature set performance for participant SHR-E. The left panel shows the pairwise comparisons for the feature sets on an individual classifier basis. The Friedman test, with a critical value of F(6,60) = 2.25 at α = 0.05, was used to determine if average accuracy ranks significantly differed from the mean rank. Classifier F Statistics (KNN: $F_F = 18.66$, SVM: $F_F = 31.62$, LDA: $F_F = 28.20$, DT: $F_F = 7.03$, RFN: $F_F = 3.70$) confirmed significant differences within each classifier's feature sets. A post-hoc Nemenyi test with a critical distance of 2.72 at α = 0.05 identified superior feature sets as indicated by pairs outside the critical distance marked by the interval bars. Each classifier is color-coded for easy comparison, with lower average ranks indicating better classification accuracy. Feature sets significantly different from the highlighted congenital feature set (CFS) were marked with an outer black ring. The right panel displays classification accuracies, which range from approximately 58% to 88%, alongside the corresponding feature sets, aligning them with the ranked performance shown in the left panel.

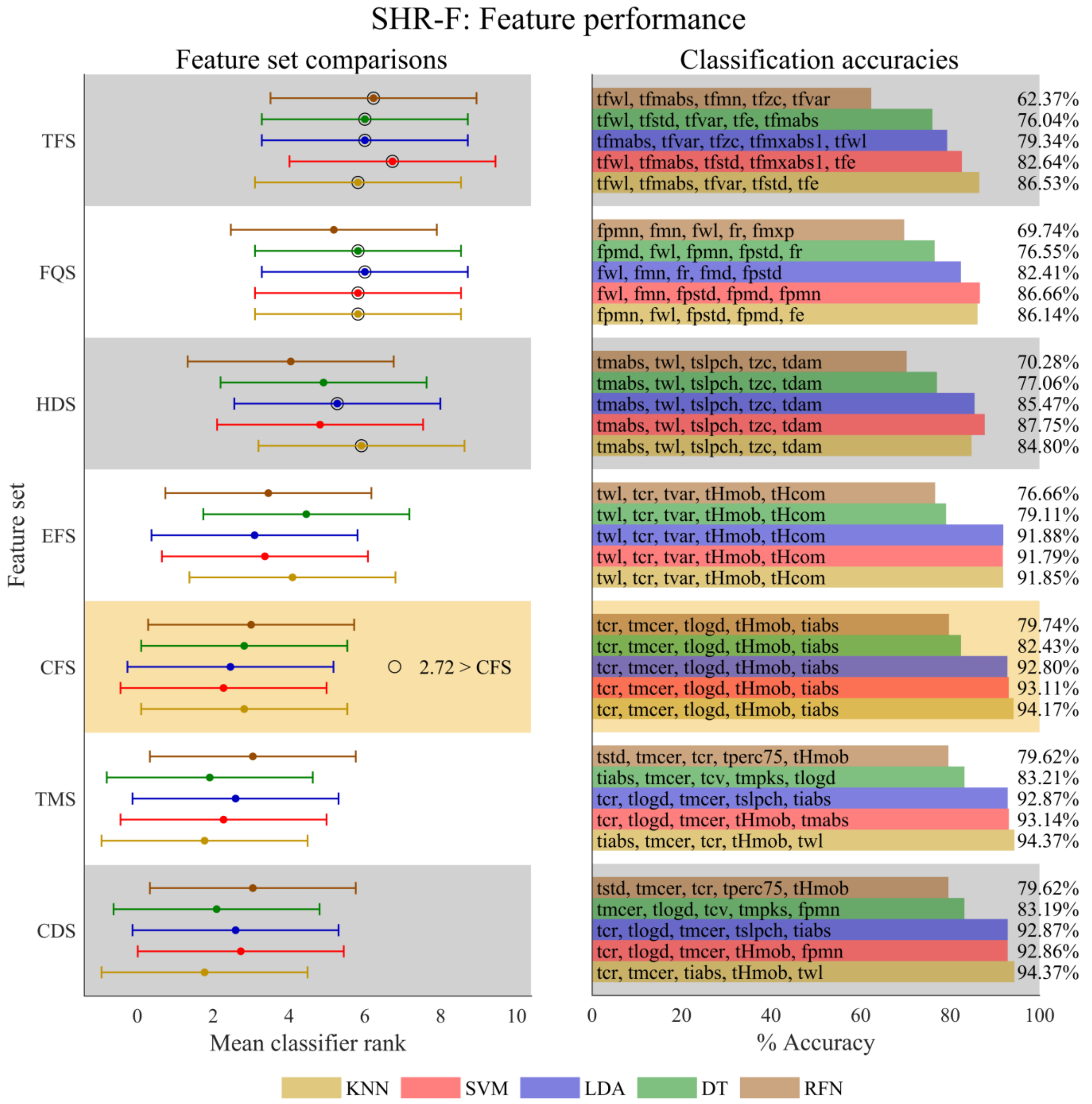

Supplementary Figure S6. Investigation of feature set performance for participant SHR-F. The left panel shows the pairwise comparisons for the feature sets on an individual classifier basis. The Friedman test, with a critical value of $F(6,60) = 2.25$ at $\alpha = 0.05$, was used to determine if average accuracy ranks significantly differed from the mean rank. Classifier F Statistics (KNN: $F_F = 33.63$, SVM: $F_F = 22.58$, LDA: $F_F = 15.01$, DT: $F_F = 17.32$, RFN: $F_F = 5.10$) confirmed significant differences within each classifier's feature sets. A post-hoc Nemenyi test with a critical distance of 2.72 at $\alpha = 0.05$ identified superior feature sets as indicated by pairs outside the critical distance marked by the interval bars. Each classifier is color-coded for easy comparison, with lower average ranks indicating better classification accuracy. Feature sets significantly different from the highlighted congenital feature set (CFS) were marked with an outer black ring. The right panel displays classification accuracies, which range from approximately 62% to 94%, alongside the corresponding feature sets, aligning them with the ranked performance shown in the left panel.

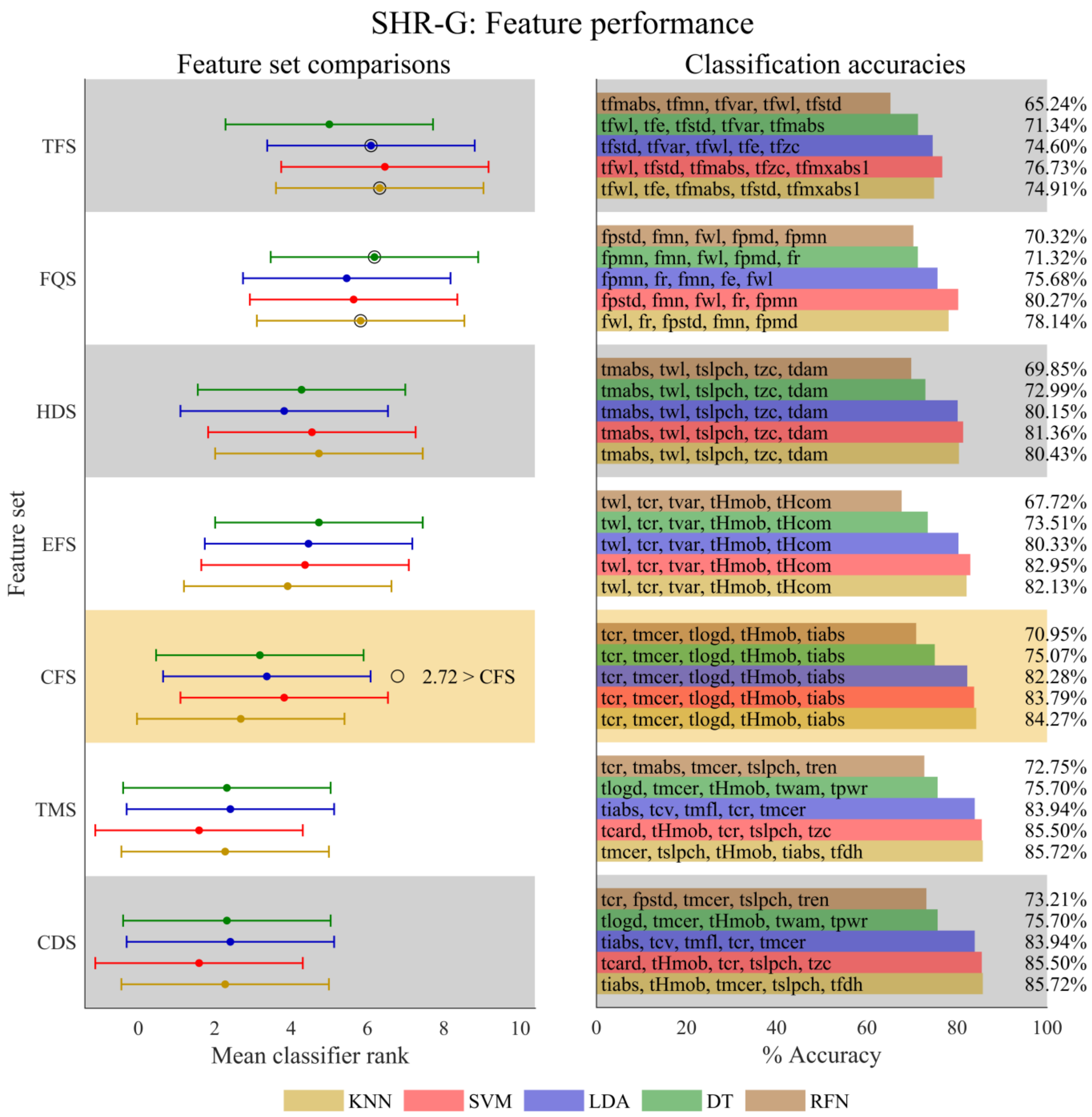

Supplementary Figure S7. Investigation of feature set performance for participant SHR-G. The left panel shows the pairwise comparisons for the feature sets on an individual classifier basis. The Friedman test, with a critical value of F(6,60) = 2.25 at α = 0.05, was used to determine if average accuracy ranks significantly differed from the mean rank. Classifier F Statistics (KNN: $F_F = 15.27$, SVM: $F_F = 28.74$, LDA: $F_F = 7.72$, DT: $F_F = 8.29$, RFN: $F_F = 1.30$) confirmed significant differences within each classifier's feature sets, with the exception of RFN. A post-hoc Nemenyi test with a critical distance of 2.72 at α = 0.05 identified superior feature sets as indicated by pairs outside the critical distance marked by the interval bars. Each classifier is color-coded for easy comparison, with lower average ranks indicating better classification accuracy. Feature sets significantly different from the highlighted congenital feature set (CFS) were marked with an outer black ring. The right panel displays classification accuracies, which range from approximately 65% to 86%, alongside the corresponding feature sets, aligning them with the ranked performance shown in the left panel.

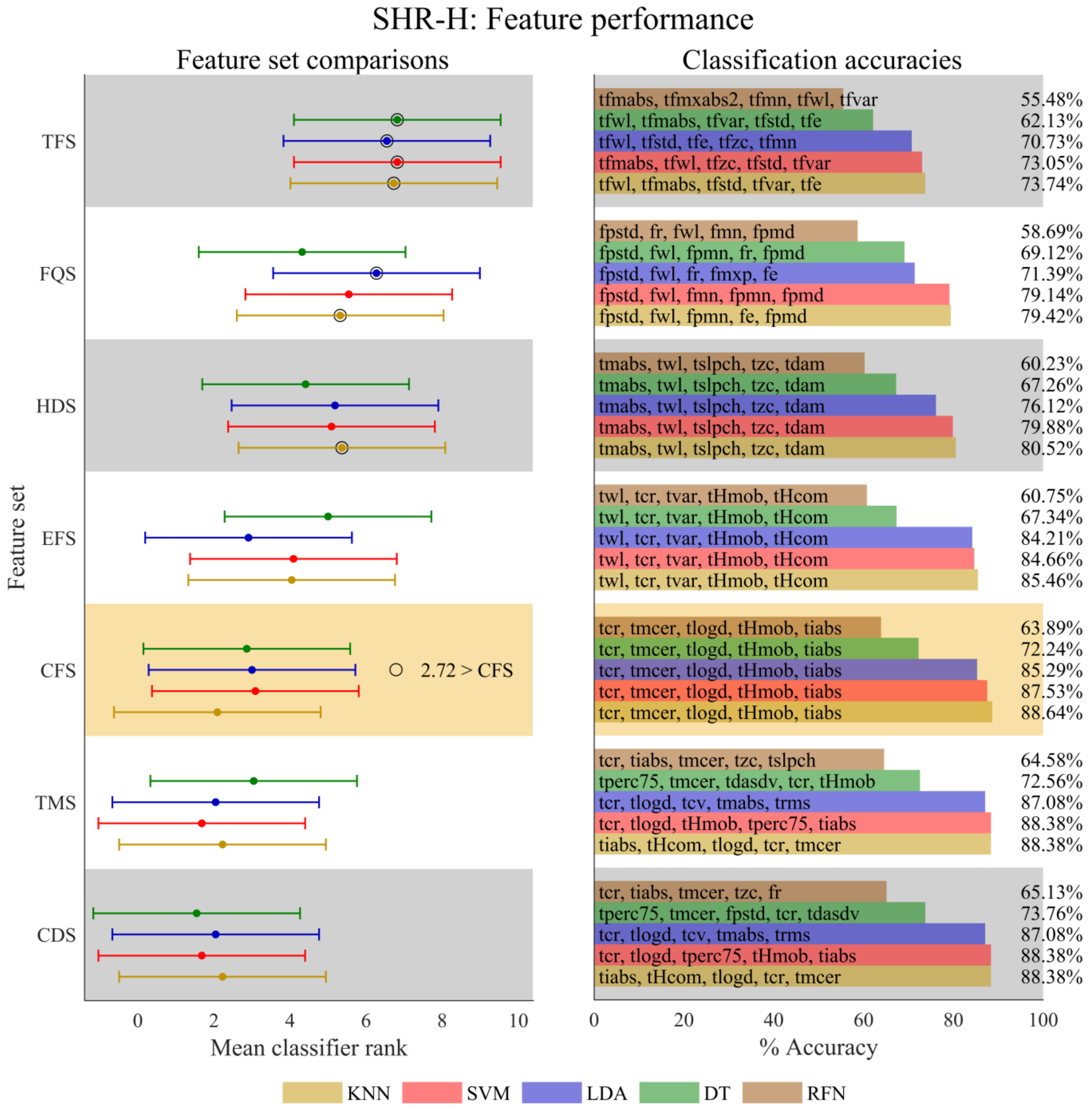

Supplementary Figure S8. Investigation of feature set performance for participant SHR-H. The left panel shows the pairwise comparisons for the feature sets on an individual classifier basis. The Friedman test, with a critical value of $F(6,60) = 2.25$ at $\alpha = 0.05$, was used to determine if average accuracy ranks significantly differed from the mean rank. Classifier F Statistics (KNN: $F_F = 29.81$, SVM: $F_F = 47.18$, LDA: $F_F = 44.60$, DT: $F_F = 16.51$, RFN: $F_F = 2.20$) confirmed significant differences within each classifier's feature sets, with the exception of RFN. A post-hoc Nemenyi test with a critical distance of 2.72 at $\alpha = 0.05$ identified superior feature sets as indicated by pairs outside the critical distance marked by the interval bars. Each classifier is color-coded for easy comparison, with lower average ranks indicating better classification accuracy. Feature sets significantly different from the highlighted congenital feature set (CFS) were marked with an outer black ring. The right panel displays classification accuracies, which range from approximately 55% to 89%, alongside the corresponding feature sets, aligning them with the ranked performance shown in the left panel.

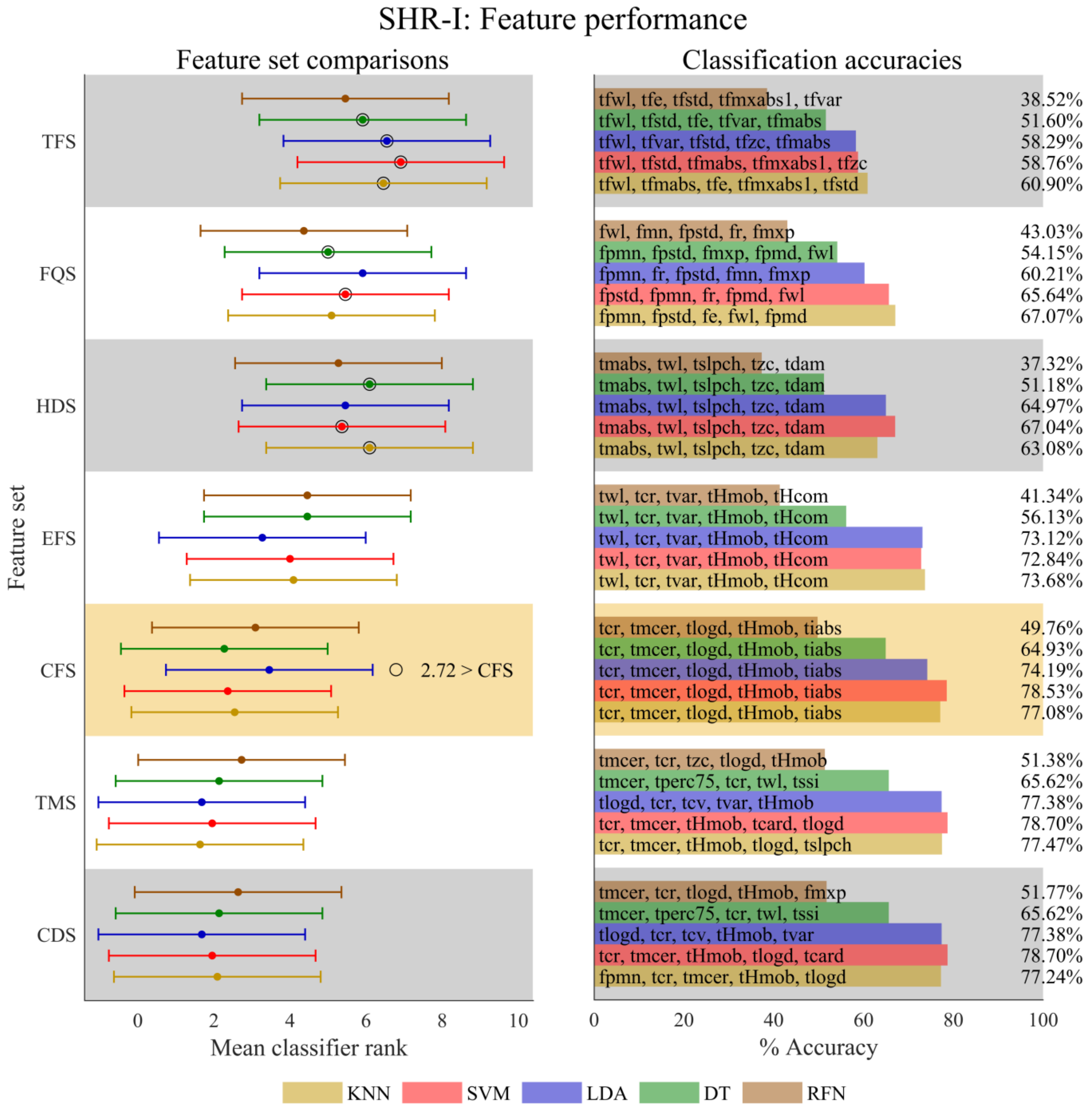

Supplementary Figure S9. Investigation of feature set performance for participant SHR-I. The left panel shows the pairwise comparisons for the feature sets on an individual classifier basis. The Friedman test, with a critical value of F(6,60) = 2.25 at $\alpha = 0.05$, was used to determine if average accuracy ranks significantly differed from the mean rank. Classifier F Statistics (KNN: $F_F = 45.36$, SVM: $F_F = 51.99$, LDA: $F_F = 56.89$, DT: $F_F = 21.63$, RFN: $F_F = 4.3$) confirmed significant differences within each classifier's feature sets. A post-hoc Nemenyi test with a critical distance of 2.72 at $\alpha = 0.05$ identified superior feature sets as indicated by pairs outside the critical distance marked by the interval bars. Each classifier is color-coded for easy comparison, with lower average ranks indicating better classification accuracy. Feature sets significantly different from the highlighted congenital feature set (CFS) were marked with an outer black ring. The right panel displays classification accuracies, which range from approximately 39% to 79%, alongside the corresponding feature sets, aligning them with the ranked performance shown in the left panel.
