# Supplementary material for: Surface electromyography evaluation for decoding hand motor intent in children with congenital upper limb deficiency
Source: Sci Rep. 2024 Dec 30;14:31741. doi: 10.1038/s41598-024-82519-z (PMC11685410; doi:10.1038/s41598-024-82519-z)
Supplement: Supplementary file 2 — Supplementary Information 2. [file 41598_2024_82519_MOESM2_ESM.pdf]

Surface electromyography evaluation for decoding hand motor intent in children with congenital upper limb deficiency

Marcus A Battraw[1], Justin Fitzgerald[2,3,4], Eden J Winslow[2], Michelle A James[5,6], Anita M Bagley[5,6], Wilsaan M Joiner[3,7], Jonathon S Schofield*[1]

[1]Department of Mechanical and Aerospace Engineering, University of California, Davis, Davis CA, USA

[2]Department of Biomedical Engineering, University of California, Davis, Davis CA, USA

[3]Department of Neurobiology, Physiology and Behavior, University of California, Davis, Davis CA, USA

[4]Clinical and Translational Science Center, University of California Davis Health, Sacramento, CA, USA

[5]Shriners Children's – Northern California, Sacramento CA, USA

[6]Department of Orthopaedic Surgery, University of California Davis Health, Sacramento CA, USA

[7]Department of Neurology, University of California Davis Health, Sacramento, CA, USA

*** Correspondence:**
Jonathon Schofield
jschofield@ucdavis.edu

Participant: SHR-A

Supplementary Table S1. SHR-A individual time domain feature performance.

Time Domain						
Feature		Classifier Accuracy				
		LDA	KNN	RFN	SVM	DT
1	tmabs	58.82	63.28	38.08	36.39	57.53
2	tstd	55.62	63.63	37.44	37.68	56.26
3	tvar	48.12	62.29	37.89	27.04	56.50
4	twl	51.64	62.71	34.65	37.47	53.58
5	trms	55.85	63.37	37.17	37.60	56.18
6	tzc	39.87	31.96	25.54	33.09	34.25
7	tpks	29.80	25.37	19.97	21.69	26.16
8	tmpks	52.20	60.12	35.45	39.35	53.81
9	tmvel	47.82	50.62	33.69	32.48	45.25
10	tslpch	40.11	35.80	26.90	28.39	35.47
11	tpwr	48.02	62.49	37.89	26.96	56.61
12	tdam	51.80	61.74	34.41	37.30	53.39
13	tmfl	51.99	62.53	34.32	36.75	53.71
14	tfd	28.25	24.21	21.70	23.95	24.25
15	tfdh	38.58	32.21	29.00	30.80	32.05
16	tren	54.92	52.85	35.53	35.51	49.66
17	tcr	60.13	59.20	37.94	39.81	52.31
18	tcv	44.93	54.75	30.80	23.55	47.03
19	tcard	58.55	59.49	32.39	39.40	52.65
20	tHmob	51.20	50.94	31.28	38.17	47.13
21	tHcom	43.57	47.44	33.61	22.65	41.62
22	tskw	31.17	34.66	18.50	14.15	32.22
23	tdasdv	51.71	61.03	33.77	38.52	54.66
24	tkurt	29.99	30.42	24.93	17.88	29.25
25	twam	49.19	51.03	28.14	30.20	47.87
26	tmcer	45.53	53.15	28.84	27.99	51.88
27	tperc75	47.41	52.92	32.08	29.30	50.68
28	tiabs	59.25	63.91	38.26	36.40	57.66
29	thist	34.39	36.17	30.19	29.89	28.06
30	tssi	47.89	63.55	37.96	27.39	56.22
31	tlogd	58.37	58.80	33.60	37.06	53.93

Supplementary Table S2. SHR-A individual frequency domain feature performance.

Frequency Domain						
Feature		Classifier Accuracy				
		LDA	KNN	RFN	SVM	DT
1	fwl	54.20	57.11	35.00	38.53	53.13
2	fmn	44.66	39.99	27.16	33.27	39.93
3	fmd	41.68	33.75	25.52	33.36	35.62
4	fpmn	52.59	53.80	30.33	26.18	46.59
5	fpmd	47.41	49.46	28.83	27.35	43.81
6	fpstd	38.48	39.48	21.19	21.15	33.53
7	fmxp	21.42	17.57	16.08	13.51	24.21
8	fr	46.79	43.27	34.18	33.62	41.54
9	fe	38.73	56.91	28.65	38.81	39.16

Supplementary Table S3. SHR-A individual time-frequency domain feature performance.

Time-Frequency Domain						
Feature		Classifier Accuracy				
		LDA	KNN	RFN	SVM	DT
1	tfstd	54.52	57.58	36.26	34.35	52.38
2	tfvar	45.15	55.85	36.23	24.26	52.41
3	tfwl	54.82	59.13	36.13	34.34	53.44
4	tfe	44.86	57.39	36.24	24.28	52.60
5	tfmxabs1	43.33	43.68	30.56	31.85	40.90
6	tfmxabs2	48.09	47.89	37.86	43.80	43.11
7	tfzc	24.75	20.30	18.30	22.01	20.98
8	tfmn	14.20	22.92	10.98	11.86	20.43
9	tfmabs	54.63	56.67	35.84	33.83	51.87

Participant: SHR-B

Supplementary Table S4. SHR-B individual time domain feature performance.

Time Domain						
Feature		Classifier Accuracy				
		LDA	KNN	RFN	SVM	DT
1	tmabs	34.41	62.53	28.05	21.73	51.05
2	tstd	34.77	62.80	25.40	22.85	51.06
3	tvar	30.43	56.48	22.33	19.26	50.94
4	twl	32.91	61.29	22.88	20.61	47.35
5	trms	35.10	63.22	25.25	22.80	50.93
6	tzc	30.69	35.45	21.87	23.52	30.71
7	tpks	28.26	38.56	20.19	22.67	31.20
8	tmpks	34.78	62.13	24.51	22.81	50.88
9	tmvel	32.94	52.72	23.15	19.60	42.74
10	tslpch	33.45	43.88	26.34	22.30	37.83
11	tpwr	30.21	57.22	22.50	19.35	51.04
12	tdam	33.04	59.74	23.21	20.46	47.48
13	tmfl	32.79	61.71	22.95	20.56	47.43
14	tfd	26.39	23.91	18.26	20.65	21.97
15	tfdh	28.99	40.01	23.05	22.07	31.45
16	tren	30.51	38.31	23.00	21.60	33.32
17	tcr	39.70	61.74	27.63	32.42	40.77
18	tcv	34.48	55.90	28.52	24.15	44.72
19	tcard	37.52	62.68	25.52	23.06	48.88
20	tHmob	33.90	54.60	23.05	26.83	40.55
21	tHcom	34.73	52.61	26.26	24.81	40.36
22	tskw	22.52	40.13	21.13	15.75	33.23
23	tdasdv	33.97	60.68	22.55	21.17	47.20
24	tkurt	18.63	35.95	18.77	13.53	26.79
25	twam	26.70	41.77	22.33	18.06	35.20
26	tmcer	28.97	55.83	25.16	19.91	45.53
27	tperc75	35.92	55.94	28.70	20.44	49.67
28	tiabs	34.37	64.48	27.55	21.88	51.30
29	thist	28.39	36.99	25.08	26.92	23.21
30	tssi	30.59	61.04	22.24	19.37	50.87
31	tlogd	39.88	61.47	28.42	21.22	49.44

Supplementary Table S5. SHR-B individual frequency domain feature performance.

Frequency Domain						
Feature		Classifier Accuracy				
		LDA	KNN	RFN	SVM	DT
1	fwl	34.90	55.18	22.38	21.00	44.32
2	fmn	31.23	41.79	19.84	24.33	34.52
3	fmd	29.61	27.65	21.13	24.69	27.65
4	fpmn	29.43	52.82	19.67	19.04	37.58
5	fpmd	26.79	51.07	19.41	18.94	38.10
6	fpstd	25.63	37.22	20.31	15.20	29.79
7	fmxp	22.67	24.81	18.67	18.47	24.96
8	fr	32.34	43.21	25.57	25.98	32.00
9	fe	24.19	53.43	20.05	20.81	33.59

Supplementary Table S6. SHR-B individual time-frequency domain feature performance.

Time-Frequency Domain						
Feature		Classifier Accuracy				
		LDA	KNN	RFN	SVM	DT
1	tfstd	34.70	57.62	26.66	22.86	47.71
2	tfvar	30.01	50.80	23.38	20.41	47.97
3	tfwl	34.38	61.08	25.59	22.59	48.05
4	tfe	29.86	55.27	23.27	20.28	47.49
5	tfmxabs1	29.58	47.88	21.67	20.96	40.32
6	tfmxabs2	33.37	50.15	24.38	26.58	39.84
7	tfzc	25.04	24.17	20.47	24.30	25.24
8	tfmn	14.91	20.28	11.78	14.16	20.20
9	tfmabs	35.46	54.66	27.61	21.85	45.88

Participant: SHR-C

Supplementary Table S7. SHR-C individual time domain feature performance.

Time Domain						
Feature		Classifier Accuracy				
		LDA	KNN	RFN	SVM	DT
1	tmabs	67.37	80.89	57.11	46.00	72.38
2	tstd	67.01	80.88	56.08	45.44	71.73
3	tvar	51.81	74.64	49.69	30.46	71.40
4	twl	68.71	85.21	55.26	44.01	72.98
5	trms	67.18	81.36	56.40	45.36	71.70
6	tzc	51.21	45.51	39.60	41.03	42.40
7	tpks	50.78	55.33	39.77	41.68	48.67
8	tmpks	65.38	79.46	55.18	43.77	69.77
9	tmvel	62.52	74.88	52.15	42.74	66.44
10	tslpch	61.10	64.64	48.34	44.44	59.34
11	tpwr	52.02	74.72	49.40	29.96	71.83
12	tdam	68.54	82.62	55.33	44.15	73.15
13	tmfl	68.58	85.11	55.23	44.53	72.91
14	tfd	33.54	27.06	23.14	27.24	25.17
15	tfdh	54.08	51.19	44.41	42.42	48.18
16	tren	56.71	53.02	43.72	44.68	52.48
17	tcr	70.93	70.25	53.86	60.00	54.66
18	tcv	53.74	67.67	44.22	40.11	63.76
19	tcard	70.64	81.45	56.18	50.97	70.30
20	tHmob	55.71	61.46	41.27	43.23	49.24
21	tHcom	57.53	73.26	42.78	46.27	60.39
22	tskw	37.48	53.96	23.82	19.53	52.32
23	tdasdv	67.86	82.15	55.68	44.05	72.46
24	tkurt	25.65	35.65	22.21	15.89	27.72
25	twam	47.06	53.39	42.58	22.13	58.79
26	tmcer	52.91	74.39	50.12	41.05	69.78
27	tperc75	63.96	74.71	56.52	45.78	71.02
28	tiabs	67.42	84.31	57.11	46.02	72.39
29	thist	36.31	37.66	35.77	37.29	27.21
30	tssi	52.44	77.82	49.67	29.89	71.76
31	tlogd	73.96	83.20	56.75	51.49	71.99

Supplementary Table S8. SHR-C individual frequency domain feature performance.

**Frequency Domain**						
**Feature**		**Classifier Accuracy**				
		**LDA**	**KNN**	**RFN**	**SVM**	**DT**
1	fwl	66.85	77.43	55.01	45.56	70.14
2	fmn	52.82	49.17	37.57	40.41	45.00
3	fmd	44.18	34.54	35.48	39.34	33.83
4	fpmn	38.21	69.81	42.85	42.08	45.08
5	fpmd	37.92	68.25	43.56	44.66	46.09
6	fpstd	35.27	55.06	38.04	35.13	35.50
7	fmxp	29.33	28.69	22.47	27.14	30.41
8	fr	52.62	52.35	43.52	39.46	44.95
9	fe	29.65	50.95	29.01	36.85	32.55

Supplementary Table S9. SHR-C individual time-frequency domain feature performance.

**Time-Frequency Domain**						
**Feature**		**Classifier Accuracy**				
		**LDA**	**KNN**	**RFN**	**SVM**	**DT**
1	tfstd	61.81	73.04	55.28	45.12	66.78
2	tfvar	48.88	66.56	46.83	29.26	66.63
3	tfwl	67.40	81.24	57.88	47.64	69.53
4	tfe	48.72	68.48	46.93	28.98	66.70
5	tfmxabs1	55.38	66.15	49.17	40.02	59.69
6	tfmxabs2	60.61	67.43	55.57	52.69	61.47
7	tfzc	30.78	22.88	23.11	29.62	23.74
8	tfmn	15.78	27.25	11.40	13.07	26.27
9	tfmabs	62.70	70.60	56.31	45.65	66.62

Participant: SHR-D

Supplementary Table S10. SHR-D individual time domain feature performance.

Time Domain						
Feature		Classifier Accuracy				
		LDA	KNN	RFN	SVM	DT
1	tmabs	44.43	49.19	22.81	39.61	43.75
2	tstd	42.71	49.15	21.70	39.50	42.58
3	tvar	39.36	48.19	22.22	36.91	42.87
4	twl	43.49	45.22	23.63	38.56	40.95
5	trms	42.66	48.73	21.82	39.00	42.96
6	tzc	34.05	28.38	26.61	36.35	27.88
7	tpks	24.56	25.26	20.39	27.10	24.40
8	tmpks	41.12	49.31	21.74	38.59	41.73
9	tmvel	41.58	42.35	23.34	33.35	37.50
10	tslpch	30.82	31.71	19.95	35.64	30.27
11	tpwr	39.70	47.93	22.19	36.51	42.65
12	tdam	43.58	45.01	23.86	38.89	40.80
13	tmfl	43.04	44.64	23.74	38.88	40.85
14	tfd	24.62	19.89	16.84	25.93	20.30
15	tfdh	28.10	26.16	20.22	28.99	23.85
16	tren	25.81	24.69	17.96	28.87	24.29
17	tcr	49.20	50.56	30.21	46.43	42.29
18	tcv	45.47	49.06	25.92	35.25	39.65
19	tcard	42.10	45.73	21.60	38.89	40.33
20	tHmob	38.85	38.30	27.81	40.30	33.86
21	tHcom	39.60	41.88	26.72	36.92	36.85
22	tskw	30.96	36.39	21.98	31.30	29.71
23	tdasdv	43.37	44.74	23.25	39.02	39.67
24	tkurt	20.44	24.58	15.83	23.04	20.27
25	twam	26.01	22.82	18.18	22.67	27.63
26	tmcer	40.76	41.74	31.93	42.55	37.84
27	tperc75	43.65	48.57	23.64	39.66	46.93
28	tiabs	44.65	49.13	22.29	40.03	43.78
29	thist	25.50	31.55	23.34	30.93	19.49
30	tssi	39.68	48.11	21.90	36.65	42.78
31	tlogd	50.66	52.45	24.66	43.08	45.39

Supplementary Table S11. SHR-D individual frequency domain feature performance.

Frequency Domain						
Feature		Classifier Accuracy				
		LDA	KNN	RFN	SVM	DT
1	fwl	40.51	41.50	21.33	38.07	38.36
2	fmn	33.47	31.71	23.72	35.28	27.86
3	fmd	26.48	18.96	20.92	26.00	19.83
4	fpmn	41.78	47.40	20.98	38.98	41.62
5	fpmd	40.22	42.96	20.87	38.72	38.83
6	fpstd	42.05	45.54	23.50	42.99	40.83
7	fmxp	28.05	25.06	19.89	29.12	25.48
8	fr	33.15	29.67	25.34	33.92	26.46
9	fe	28.18	42.71	19.91	35.90	27.09

Supplementary Table S12. SHR-D individual time-frequency domain feature performance.

Time-Frequency Domain						
Feature		Classifier Accuracy				
		LDA	KNN	RFN	SVM	DT
1	tfstd	41.64	40.87	22.72	40.78	37.32
2	tfvar	37.81	40.42	21.02	37.49	37.30
3	tfwl	41.28	41.65	22.52	38.88	38.81
4	tfe	37.33	40.19	20.97	37.25	36.93
5	tfmxabs1	34.68	37.33	19.82	36.75	32.37
6	tfmxabs2	37.41	38.29	27.02	39.32	31.20
7	tfzc	26.19	18.83	18.44	26.71	19.52
8	tfmn	11.62	17.44	10.35	18.80	17.15
9	tfmabs	43.70	40.83	23.92	42.00	37.78

Participant: SHR-E

Supplementary Table S13. SHR-E individual time domain feature performance.

Time Domain						
Feature		Classifier Accuracy				
		LDA	KNN	RFN	SVM	DT
1	tmabs	69.64	77.50	52.21	71.72	70.11
2	tstd	65.86	75.12	50.93	67.99	67.25
3	tvar	46.64	69.97	43.56	59.43	67.13
4	twl	68.18	75.90	49.39	70.69	66.56
5	trms	65.53	75.35	51.16	67.89	67.37
6	tzc	62.10	52.40	41.15	60.67	50.39
7	tpks	49.84	49.67	39.49	51.12	42.58
8	tmpks	59.83	71.26	47.05	64.43	64.26
9	tmvel	59.52	66.44	45.01	61.64	61.36
10	tslpch	66.11	60.72	43.81	65.22	57.26
11	tpwr	46.75	69.70	43.79	59.91	66.91
12	tdam	68.27	75.10	49.83	70.29	66.27
13	tmfl	68.42	75.77	49.74	70.63	66.56
14	tfd	40.17	31.97	27.29	40.96	31.02
15	tfdh	58.56	54.36	41.53	57.82	49.44
16	tren	52.72	49.44	36.28	55.84	46.76
17	tcr	70.41	72.19	54.62	74.30	55.96
18	tcv	52.11	61.88	40.65	58.49	56.82
19	tcard	73.81	75.66	54.43	71.47	66.78
20	tHmob	61.80	65.40	44.70	63.75	57.44
21	tHcom	59.06	62.01	42.78	59.42	51.87
22	tskw	36.71	58.49	29.36	41.61	54.15
23	tdasdv	64.91	72.62	48.52	67.22	65.26
24	tkurt	23.23	34.14	23.19	27.97	27.45
25	twam	40.39	46.08	30.32	40.37	48.11
26	tmcer	45.35	67.04	39.98	59.48	60.76
27	tperc75	65.58	70.17	49.07	69.54	67.56
28	tiabs	69.51	80.28	51.72	71.53	69.71
29	thist	44.93	44.54	43.56	49.81	34.11
30	tssi	46.55	73.36	43.89	59.89	67.11
31	tlogd	78.09	79.24	53.47	75.69	72.16

Supplementary Table S14. SHR-E individual frequency domain feature performance.

Frequency Domain						
Feature		Classifier Accuracy				
		LDA	KNN	RFN	SVM	DT
1	fwl	69.48	72.13	49.16	71.76	67.34
2	fmn	57.29	56.68	41.51	58.01	50.86
3	fmd	49.25	39.07	34.65	49.50	39.22
4	fpmn	64.71	73.40	50.17	67.60	66.48
5	fpmd	64.56	72.06	48.74	66.67	65.42
6	fpstd	62.44	70.74	50.34	68.60	67.35
7	fmxp	31.03	28.12	23.38	33.08	26.81
8	fr	54.50	57.33	39.32	57.46	50.30
9	fe	36.02	50.37	25.81	45.56	38.46

Supplementary Table S15. SHR-E individual time-frequency domain feature performance.

Time-Frequency Domain						
Feature		Classifier Accuracy				
		LDA	KNN	RFN	SVM	DT
1	tfstd	63.09	71.05	49.63	67.55	65.59
2	tfvar	44.22	64.56	42.79	57.71	65.47
3	tfwl	70.39	76.57	51.53	71.71	69.09
4	tfe	43.88	67.91	42.37	57.43	65.04
5	tfmxabs1	53.33	60.24	44.21	57.83	55.37
6	tfmxabs2	56.06	59.46	49.73	60.52	54.40
7	tfzc	36.18	25.97	25.49	35.36	26.07
8	tfmn	15.48	25.23	10.88	19.57	23.46
9	tfmabs	65.46	71.70	50.34	69.77	67.98

Participant: SHR-F

Supplementary Table S16. SHR-F individual time domain feature performance.

Time Domain						
Feature		Classifier Accuracy				
		LDA	KNN	RFN	SVM	DT
1	tmabs	70.60	88.41	57.95	79.70	79.05
2	tstd	70.43	87.24	61.50	79.41	78.67
3	tvar	56.72	82.91	56.65	71.50	79.19
4	twl	69.64	88.24	55.95	77.74	77.87
5	trms	70.49	87.62	61.36	79.44	78.47
6	tzc	60.06	52.02	45.29	61.52	47.49
7	tpks	65.75	69.75	44.02	68.19	59.96
8	tmpks	64.86	84.90	54.96	77.92	77.56
9	tmvel	65.08	80.61	55.15	70.94	71.24
10	tslpch	75.81	70.56	50.63	74.43	61.39
11	tpwr	56.94	83.08	56.38	71.50	78.16
12	tdam	69.69	86.26	55.98	78.04	77.97
13	tmfl	69.89	88.34	55.71	78.01	78.04
14	tfd	45.26	35.95	29.25	44.47	32.84
15	tfdh	72.58	66.15	52.33	71.62	58.54
16	tren	46.87	46.78	36.84	52.63	43.43
17	tcr	82.70	85.74	61.12	85.51	74.02
18	tcv	64.33	82.98	48.79	72.43	74.70
19	tcard	78.33	87.94	58.08	80.91	76.49
20	tHmob	71.03	72.53	53.41	71.11	59.23
21	tHcom	70.78	76.84	52.71	72.13	66.96
22	tskw	47.59	65.50	20.59	53.04	66.28
23	tdasdv	69.21	84.91	54.10	77.80	77.99
24	tkurt	38.30	55.90	26.56	44.18	47.61
25	twam	42.07	48.62	23.62	40.45	49.15
26	tmcer	66.71	85.19	58.03	77.88	79.50
27	tperc75	67.48	80.17	54.43	74.90	75.40
28	tiabs	70.58	90.55	58.03	79.29	79.55
29	thist	52.46	53.27	50.05	57.12	43.18
30	tssi	56.68	85.17	56.40	71.46	78.40
31	tlogd	79.29	87.86	56.82	82.42	77.33

Supplementary Table S17. SHR-F individual frequency domain feature performance.

Frequency Domain						
Feature		Classifier Accuracy				
		LDA	KNN	RFN	SVM	DT
1	fwl	70.22	84.34	57.04	78.62	76.21
2	fmn	65.44	61.18	51.58	66.74	53.96
3	fmd	55.08	42.70	40.80	55.00	39.97
4	fpmn	70.18	86.37	60.71	78.96	77.69
5	fpmd	68.88	84.84	59.97	78.82	76.84
6	fpstd	69.07	80.18	57.35	76.52	72.46
7	fmxp	41.03	37.55	33.04	43.01	37.46
8	fr	63.30	65.57	54.68	65.77	55.25
9	fe	41.94	61.06	26.06	57.54	46.53

Supplementary Table S18. SHR-F individual time-frequency domain feature performance.

Time-Frequency Domain						
Feature		Classifier Accuracy				
		LDA	KNN	RFN	SVM	DT
1	tfstd	68.14	83.26	59.53	77.67	74.21
2	tfvar	53.92	78.46	53.91	67.20	74.24
3	tfwl	69.98	88.12	59.58	79.56	77.31
4	tfe	53.72	80.38	53.41	67.39	73.51
5	tfmxabs1	61.84	74.23	51.44	71.50	66.04
6	tfmxabs2	64.69	72.40	55.01	72.44	65.14
7	tfzc	37.48	27.59	26.88	37.66	28.30
8	tfmn	13.75	25.55	9.79	22.56	23.30
9	tfmabs	68.84	82.27	57.89	77.61	73.27

Participant: SHR-G

Supplementary Table S19. SHR-G individual time domain feature performance.

Time Domain						
Feature		Classifier Accuracy				
		LDA	KNN	RFN	SVM	DT
1	tmabs	69.47	76.52	44.35	74.01	72.72
2	tstd	66.70	75.50	41.49	73.08	71.29
3	tvar	49.88	71.55	35.28	57.64	71.32
4	twl	63.72	76.38	38.38	66.25	68.75
5	trms	66.73	75.20	41.67	73.19	71.42
6	tzc	60.62	57.00	44.10	61.23	53.36
7	tpks	52.73	51.16	38.82	54.98	47.64
8	tmpks	60.47	73.33	37.41	66.12	70.56
9	tmvel	59.05	69.07	37.63	63.24	64.79
10	tslpch	62.76	61.27	36.58	65.63	58.28
11	tpwr	49.90	71.53	35.74	57.72	71.56
12	tdam	63.45	73.21	38.17	66.27	68.72
13	tmfl	64.02	76.33	38.14	66.43	68.50
14	tfd	43.19	38.94	32.95	44.26	34.86
15	tfdh	57.10	57.74	43.32	58.69	52.62
16	tren	63.90	63.23	42.50	66.23	60.05
17	tcr	71.44	72.14	55.00	72.69	64.10
18	tcv	55.18	71.76	39.24	67.53	68.83
19	tcard	75.15	78.51	39.34	77.09	72.11
20	tHmob	58.20	64.25	43.98	62.19	55.59
21	tHcom	57.05	63.67	40.84	60.77	56.36
22	tskw	40.48	56.59	30.37	43.50	57.64
23	tdasdv	60.91	72.97	35.56	65.47	67.23
24	tkurt	25.99	41.57	16.61	31.89	35.56
25	twam	57.90	59.05	23.69	60.21	62.61
26	tmcer	55.44	69.84	50.49	66.67	67.34
27	tperc75	65.93	71.21	46.01	70.61	69.54
28	tiabs	69.55	79.07	44.58	74.02	72.56
29	thist	46.67	47.59	43.08	50.65	36.95
30	tssi	49.64	76.25	35.00	57.78	71.17
31	tlogd	75.93	77.07	42.92	76.00	72.58

Supplementary Table S20. SHR-G individual frequency domain feature performance.

**Frequency Domain**						
**Feature**		**Classifier Accuracy**				
		**LDA**	**KNN**	**RFN**	**SVM**	**DT**
1	fwl	66.91	72.55	39.76	72.56	70.07
2	fmn	53.20	55.46	41.93	56.96	51.13
3	fmd	50.04	45.12	39.36	51.77	42.68
4	fpmn	66.41	74.75	41.44	73.34	71.34
5	fpmd	64.85	72.40	38.51	71.49	69.39
6	fpstd	67.66	72.13	46.08	73.70	70.90
7	fmxp	32.50	29.32	24.95	34.15	30.74
8	fr	58.66	61.29	43.87	61.12	51.89
9	fe	35.43	61.47	28.35	57.30	52.82

Supplementary Table S21. SHR-G individual time-frequency domain feature performance.

**Time-Frequency Domain**						
**Feature**		**Classifier Accuracy**				
		**LDA**	**KNN**	**RFN**	**SVM**	**DT**
1	tfstd	65.91	72.90	42.17	71.60	69.83
2	tfvar	49.11	68.45	38.44	57.60	69.83
3	tfwl	69.06	76.60	44.24	73.56	71.92
4	tfe	48.97	72.96	37.83	57.32	69.77
5	tfmxabs1	55.31	67.67	36.58	62.10	64.58
6	tfmxabs2	57.65	64.76	41.66	65.80	63.21
7	tfzc	37.64	29.43	28.67	37.86	29.97
8	tfmn	22.76	35.17	12.67	26.35	35.06
9	tfmabs	67.89	73.08	44.07	72.77	70.91

Participant: SHR-H

Supplementary Table S22. SHR-H individual time domain feature performance.

Time Domain						
Feature		Classifier Accuracy				
		LDA	KNN	RFN	SVM	DT
1	tmabs	66.12	80.02	48.77	67.88	66.46
2	tstd	63.29	78.32	46.71	63.62	63.07
3	tvar	56.80	75.81	46.18	61.63	62.65
4	twl	57.07	66.35	36.50	53.55	52.85
5	trms	63.05	77.98	46.44	63.46	62.76
6	tzc	47.68	44.54	36.39	50.36	39.38
7	tpks	39.23	35.93	28.85	39.32	32.15
8	tmpks	57.64	70.77	42.41	58.32	58.19
9	tmvel	52.93	55.03	31.83	48.10	45.77
10	tslpch	47.04	45.04	32.65	49.45	40.91
11	tpwr	56.85	75.75	46.03	61.47	62.97
12	tdam	56.87	65.72	36.32	53.33	52.63
13	tmfl	56.87	66.52	36.20	53.35	52.52
14	tfd	32.99	26.67	23.16	33.73	25.12
15	tfdh	46.30	40.41	34.83	45.66	36.22
16	tren	32.78	29.88	22.72	37.07	30.32
17	tcr	74.45	75.99	48.88	74.99	59.66
18	tcv	72.07	74.61	42.02	66.95	61.04
19	tcard	61.48	70.57	42.84	59.70	56.95
20	tHmob	53.28	59.05	35.53	54.82	45.23
21	tHcom	51.71	58.96	36.16	51.47	47.83
22	tskw	33.60	39.44	24.24	34.43	33.77
23	tdasdv	54.34	63.86	36.62	53.24	52.41
24	tkurt	32.53	39.88	29.28	36.06	32.89
25	twam	23.51	21.05	18.50	23.76	27.15
26	tmcer	54.72	64.82	42.13	61.44	56.93
27	tperc75	63.01	74.22	44.67	68.03	66.16
28	tiabs	66.24	81.02	48.32	67.60	66.08
29	thist	36.40	38.19	34.65	42.52	28.70
30	tssi	56.68	76.24	46.05	61.51	62.43
31	tlogd	69.70	79.47	46.62	70.69	67.82

Supplementary Table S23. SHR-H individual frequency domain feature performance.

Frequency Domain						
Feature		Classifier Accuracy				
		LDA	KNN	RFN	SVM	DT
1	fwl	55.54	59.21	38.26	56.04	50.78
2	fmn	43.38	40.98	27.74	45.64	36.21
3	fmd	37.12	29.31	27.41	38.17	29.97
4	fpmn	54.40	72.51	41.55	62.40	59.95
5	fpmd	53.22	63.96	40.82	56.99	53.75
6	fpstd	56.08	72.78	41.49	64.96	65.14
7	fmxp	31.31	29.17	22.85	32.39	31.89
8	fr	41.12	40.30	33.90	41.52	34.08
9	fe	44.20	59.53	30.33	51.32	40.03

Supplementary Table S24. SHR-H individual time-frequency domain feature performance.

Time-Frequency Domain						
Feature		Classifier Accuracy				
		LDA	KNN	RFN	SVM	DT
1	tfstd	59.95	69.26	45.44	63.09	59.65
2	tfvar	52.96	66.92	43.06	59.53	59.33
3	tfwl	62.84	71.30	46.23	63.51	60.32
4	tfe	52.36	66.48	43.02	59.01	58.72
5	tfmxabs1	46.29	47.08	32.97	48.58	41.52
6	tfmxabs2	51.52	51.33	41.60	54.83	43.94
7	tfzc	37.03	29.97	26.81	37.67	29.76
8	tfmn	16.81	19.88	13.72	19.64	19.04
9	tfmabs	60.11	68.59	46.92	64.10	59.83

Participant: SHR-I

Supplementary Table S25. SHR-I individual time domain feature performance.

Time Domain						
Feature		Classifier Accuracy				
		LDA	KNN	RFN	SVM	DT
1	tmabs	52.58	66.09	35.72	52.10	54.90
2	tstd	52.75	65.38	35.77	51.76	54.60
3	tvar	37.60	62.79	36.81	44.82	55.04
4	twl	49.38	60.89	38.10	48.37	53.35
5	trms	52.96	65.67	35.76	51.36	54.99
6	tzc	40.54	35.22	31.27	42.11	32.31
7	tpks	37.12	38.04	29.70	37.42	34.01
8	tmpks	50.81	61.71	35.15	50.74	52.55
9	tmvel	45.77	55.66	35.19	44.07	49.90
10	tslpch	40.13	41.85	33.47	38.66	37.91
11	tpwr	37.04	62.16	36.66	45.22	54.93
12	tdam	49.17	62.09	38.15	48.28	52.82
13	tmfl	49.23	60.48	38.34	48.10	53.03
14	tfd	28.47	24.29	20.49	29.41	22.95
15	tfdh	39.19	36.96	31.61	41.36	34.18
16	tren	33.80	32.42	29.01	35.88	35.19
17	tcr	56.96	61.88	40.03	60.69	47.61
18	tcv	45.73	60.97	26.48	49.61	50.18
19	tcard	56.96	58.13	33.60	51.43	53.12
20	tHmob	42.41	47.22	31.19	45.95	37.79
21	tHcom	35.71	48.56	33.13	39.18	38.54
22	tskw	33.26	46.31	25.32	38.29	41.46
23	tdasdv	48.66	62.32	37.48	46.96	53.12
24	tkurt	23.16	31.63	16.80	26.88	24.92
25	twam	36.20	30.33	25.66	35.40	39.31
26	tmcer	44.22	59.65	41.27	59.25	56.14
27	tperc75	46.42	52.05	35.06	49.04	50.83
28	tiabs	52.03	64.81	36.01	52.11	55.33
29	thist	31.40	34.13	28.93	35.51	24.54
30	tssi	37.04	64.21	36.65	45.52	54.59
31	tlogd	57.94	63.43	34.76	54.41	53.40

Supplementary Table S26. SHR-I individual frequency domain feature performance.

Frequency Domain						
Feature		Classifier Accuracy				
		LDA	KNN	RFN	SVM	DT
1	fwl	50.15	56.14	37.41	49.83	50.33
2	fmn	38.69	37.99	29.25	40.43	33.04
3	fmd	29.09	21.19	23.58	29.00	21.61
4	fpmn	52.61	63.26	35.24	52.23	53.18
5	fpmd	50.43	58.27	34.38	50.50	50.24
6	fpstd	52.56	60.70	36.90	53.80	51.22
7	fmxp	22.60	24.56	19.48	25.02	24.63
8	fr	37.80	38.06	30.83	39.09	31.33
9	fe	25.13	45.05	20.15	29.32	28.22

Supplementary Table S27. SHR-I individual time-frequency domain feature performance.

Time-Frequency Domain						
Feature		Classifier Accuracy				
		LDA	KNN	RFN	SVM	DT
1	tfstd	51.82	58.24	34.69	51.00	50.33
2	tfvar	35.74	55.48	34.89	43.80	51.33
3	tfwl	52.32	59.28	38.25	52.20	53.02
4	tfe	35.23	56.72	34.58	43.56	50.20
5	tfmxabs1	44.96	48.06	31.82	45.47	42.78
6	tfmxabs2	42.65	45.78	32.91	46.03	40.75
7	tfzc	23.41	19.42	17.65	23.42	19.33
8	tfmn	11.46	17.39	9.44	16.58	19.02
9	tfmabs	49.38	55.74	35.39	50.63	49.84
